# A phosphorylation-deficient mutant of *Sik3*, a homolog of *Sleepy*, alters circadian sleep regulation by PDF neurons in *Drosophila*

**DOI:** 10.3389/fnins.2023.1181555

**Published:** 2023-08-17

**Authors:** Riho Kobayashi, Shin Nakane, Jun Tomita, Hiromasa Funato, Masashi Yanagisawa, Kazuhiko Kume

**Affiliations:** ^1^Department of Neuropharmacology, Graduate School of Pharmaceutical Sciences, Nagoya City University, Nagoya, Japan; ^2^International Institute for Integrative Sleep Medicine (WPI-IIIS), University of Tsukuba, Tsukuba, Japan; ^3^School of Medicine, Toho University, Tokyo, Japan

**Keywords:** sleep, PDF, *Sik3*, circadian rhythm, *Drosophila*

## Abstract

Sleep behavior has been observed from non-vertebrates to humans. *Sleepy* mutation in mice resulted in a notable increase in sleep and was identified as an exon-skipping mutation of the *salt-inducible kinase 3 (Sik3)* gene, conserved among animals. The skipped exon includes a serine residue that is phosphorylated by protein kinase A. Overexpression of a mutant gene with the conversion of this serine into alanine (*Sik3-SA*) increased sleep in both mice and the fruit fly *Drosophila melanogaster*. However, the mechanism by which *Sik3-SA* increases sleep remains unclear. Here, we found that *Sik3-SA* overexpression in all neurons increased sleep under both light–dark (LD) conditions and constant dark (DD) conditions in *Drosophila*. Additionally, overexpression of *Sik3-SA* only in PDF neurons, which are a cluster of clock neurons regulating the circadian rhythm, increased sleep during subjective daytime while decreasing the amplitude of circadian rhythm. Furthermore, suppressing *Sik3-SA* overexpression specifically in PDF neurons in flies overexpressing *Sik3-SA* in all neurons reversed the sleep increase during subjective daytime. These results indicate that *Sik3-SA* alters the circadian function of PDF neurons and leads to an increase in sleep during subjective daytime under constant dark conditions.

## Highlights

- *Sik3-SA* overexpression in all neurons increased sleep in *Drosophila.*
- *Sik3-SA* overexpression altered the circadian sleep regulation by PDF neurons in *Drosophila.*
- *Sik3-SA* overexpression in PDF neurons reduced the circadian amplitude of the activity under DD condition.
- PDF neurons regulate the circadian rhythmicity of sleep under DD condition.

## Introduction

Sleep is a conserved phenomenon observed across various species. In humans, it is hypothesized that sleep is regulated by two processes: circadian rhythm (referred to as process C) and sleep homeostasis (referred to as process S). These processes independently contribute to the arousal threshold and sleepiness, determining the timing and duration of daily sleep (Borbély et al., 2016). While growing evidence suggests that these processes mutually influence each other and are inseparable, the underlying molecular mechanisms regulating their interaction remain elusive.

*Drosophila melanogaster*, with its extensive genetic toolkit, has been widely employed as a versatile model organism for studying sleep (Hendricks et al., 2000; Shaw et al., 2000). Our previous studies have highlighted similarities in the molecular basis of sleep regulation, arousal, and circadian rhythms between mammals and *Drosophila melanogaster* (Tomita et al., 2011, 2017; Ueno et al., 2012; Hasegawa et al., 2017; Nakagawa et al., 2022a,b; Yamaguchi et al., 2022a,b). Through a large-scale forward genetic screening of sleep behaviors in ethylnitrosourea-mutagenized mice, we identified a mutation in *the salt-inducible kinase 3 (Sik3)* gene that increased non rapid-eye movement (NREM) sleep, which we named *Sleepy* (Funato et al., 2016). SIK3 is a serine–threonine kinase belonging to the AMP-dependent protein kinase (AMPK) family, involved in the intracellular signal transduction and lipid metabolism (Choi et al., 2015). Our investigations revealed that the *Sleepy* mutation in mice resulted from an exon skipping splicing mutation, leading to the deletion of 52 amino acids in exon 13 (ΔEx13). This exon contains the RRAS sequence, which serves as recognition site for protein kinase A (PKA) phosphorylation at the serine (S) residue. Notably, this RRAS sequence and its surrounding amino acid sequence are highly conserved not only in vertebrates but also in invertebrates like *Caenorhabditis elegans* and *Drosophila melanogaster*. Given that sleep is observed across diverse animal species, we hypothesized that phosphorylation of this serine residue plays a crucial role in sleep regulation. To test this hypothesis, we analyzed a phosphorylation-deficient mutant form of the *Sik3* gene, which we refer *Sik3-SA*. In this mutant, the serine residue at position 563 in *Drosophila* and 551 in mice, is replaced with alanine. Our findings revealed that pan-neuronal overexpression of *Sik3-SA (S563A)* increased sleep in *Drosophila*, and the replacement of one allele of the normal gene with *Sik3-SA (S551A)* increased sleep in mice (Funato et al., 2016; Honda et al., 2018). Moreover, endogenous *Sik3* has been reported to regulate circadian behavior in both flies and mice (Fujii et al., 2017; Hayasaka et al., 2017). In the present study, we investigate the role of *Sik3-SA* in sleep regulation in *D. melanogaster* using genetic approaches.

## Material and methods

### Fly strains and culture conditions

**Figure 1:**
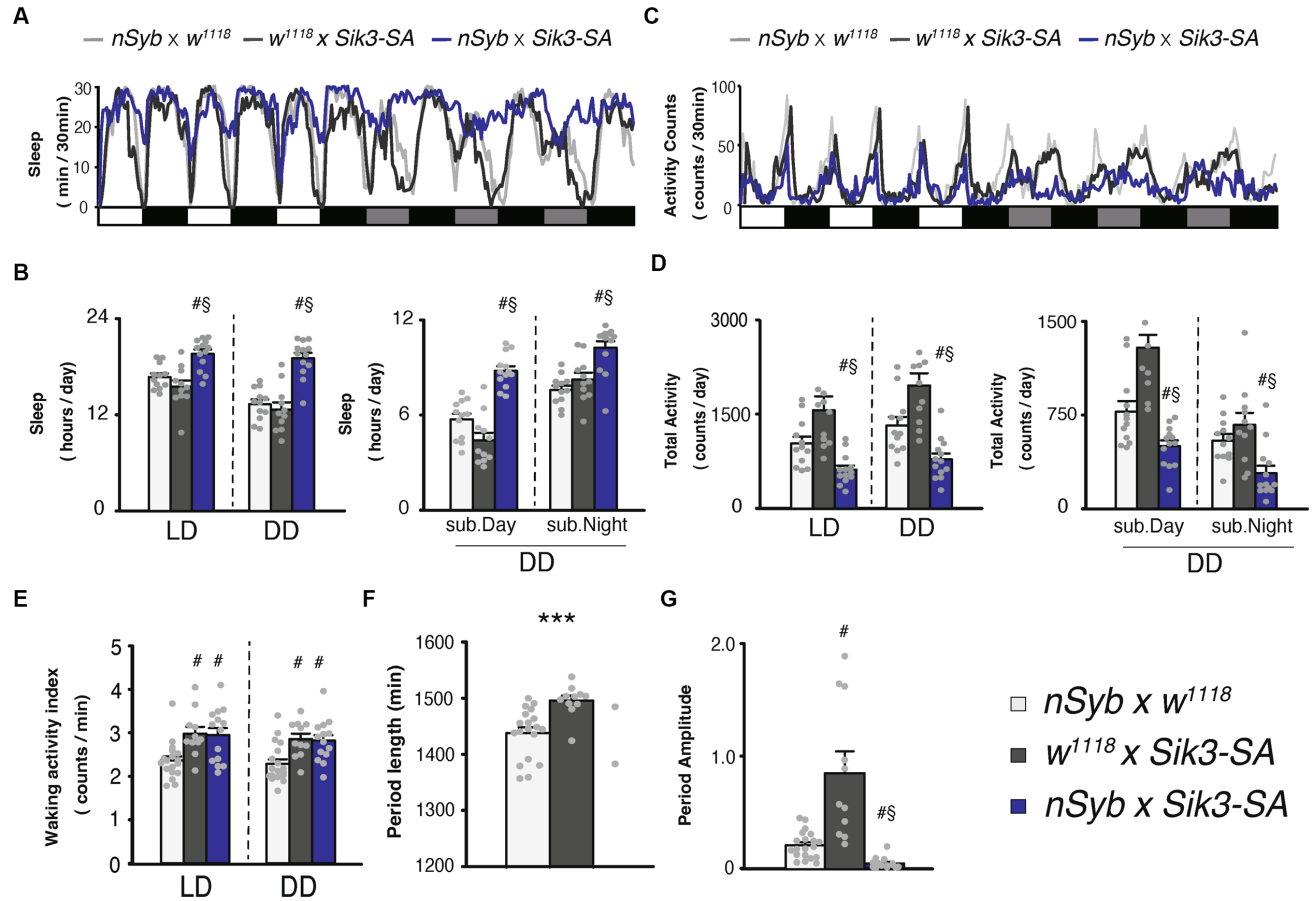
Pan-neuronal *Sik3-SA* overexpression increased sleep, particularly under DD conditions. **(A)** Average sleep profiles for 30-min intervals of control flies (*nSyb*-*GAL4* × *w^1118^,* light gray line, *n* = 19 and *w^1118^* × *UAS*-*Sik3-SA,* dark gray line, *n* = 11) and flies overexpressing *Sik3-SA* in all neurons (*nSyb*-*GAL4* × *UAS*-*Sik3-SA*, blue line, *n* = 14). Each fly was recorded for 3 days under LD followed by 3 days under constant dark (DD) conditions. Day and night under LD and subjective day and night under DD are depicted by white, black, gray, and black bars, respectively. **(B)** Total daily sleep of *nSyb*-*GAL4* × *w^1118^* (light gray bar), *w^1118^* × *UAS*-*Sik3-SA* (dark gray bar) and *nSyb*-*GAL4* × *UAS*-*Sik3-SA* (blue bar) flies under LD and DD (left) and during subjective daytime and nighttime under DD condition (right). **(C)** Average active counts profiles for 30-min intervals of control flies (*nSyb*-*GAL4* × *w^1118^,* light gray line, *n* = 19 and *w^1118^* × *UAS*-*Sik3-SA,* dark gray line, *n* = 11) and flies overexpressing *Sik3-SA* in all neurons (*nSyb*-*GAL4* × *UAS*-*Sik3-SA*, blue line, *n* = 14). **(D)** Total activity counts of *nSyb*-*GAL4* × *w^1118^* (light gray bar), *w^1118^* × *UAS*-*Sik3-SA* (dark gray bar) and *nSyb*-*GAL4* × *UAS*-*Sik3-SA* (blue bar) flies under LD and DD condition (left) and during subjective daytime and nighttime under DD condition (right). **(E)** Waking activity index of *nSyb*-*GAL4* × *w^1118^* (light gray bar), *w^1118^* × *UAS*-*Sik3-SA* (dark gray bar) and *nSyb*-*GAL4* × *UAS*-*Sik3-SA* (blue bar) flies under LD and DD. **(F)** The period length of *nSyb*-*GAL4* × *w^1118^* (light gray bar, *n* = 19), *w^1118^* × *UAS*-*Sik3-SA* (dark gray bar, *n* = 11) and *nSyb*-*GAL4* × *UAS*-*Sik3-SA* (just two plots, *n* = 2). **(G)** The circadian period amplitude of *nSyb*-*GAL4* × *w^1118^* (light gray bar, *n* = 19), *w^1118^* × *UAS*-*Sik3-SA* (dark gray bar, *n* = 11) and *nSyb*-*GAL4* × *UAS*-*Sik3-SA* (blue bar, *n* = 14). Data are presented as mean ± SEM. #*p* < 0.05 vs. *nSyb*-*GAL4* × *w^1118^*, §:*p* < 0.05 vs. *w^1118^* × *UAS*-*Sik3-SA*; Kruskal-Wallis test followed by Dunn-Bonferroni pairwise comparisons; ***p < 0.001; Welch’s *t*-test.

**Figure 2:**
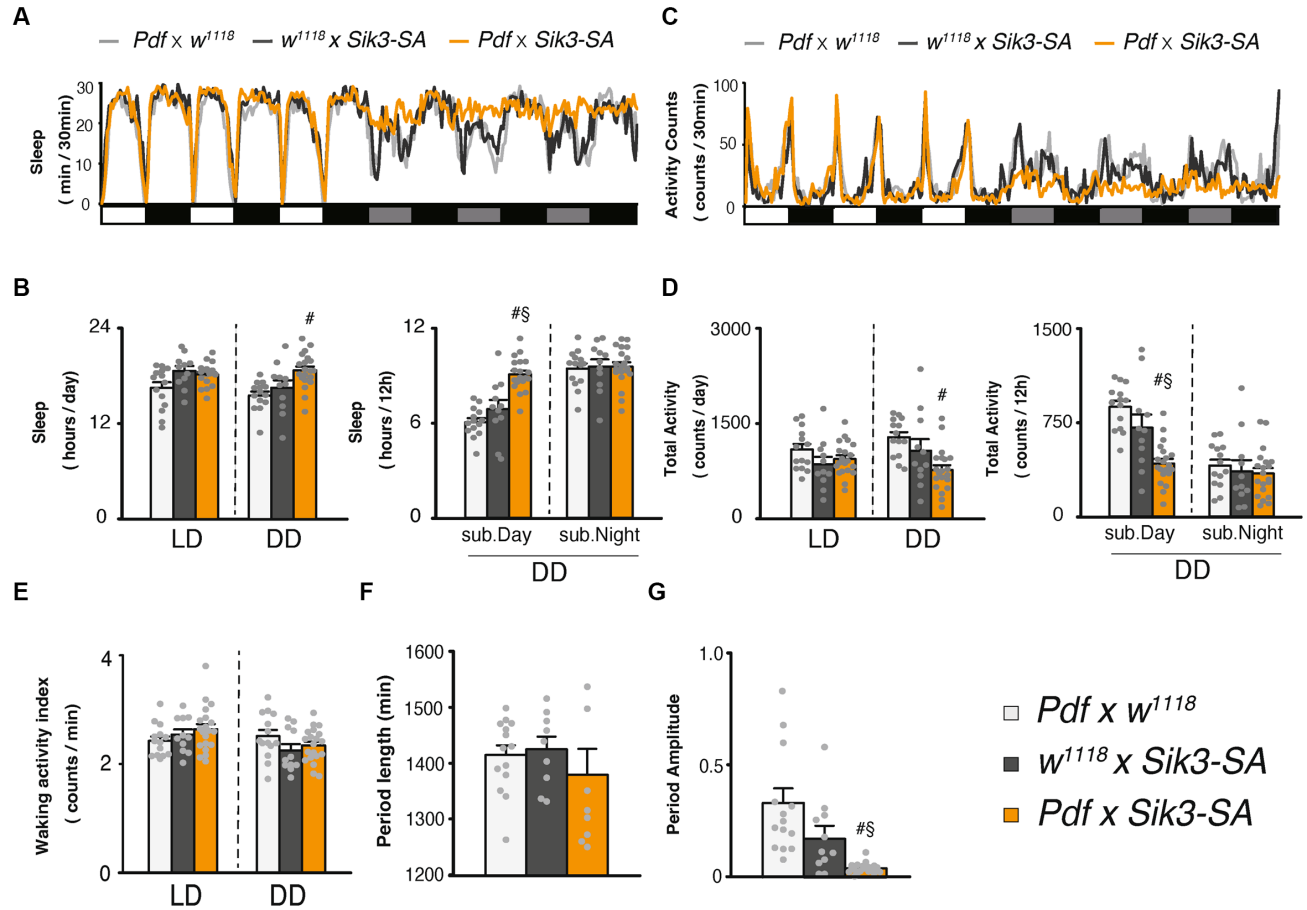
Overexpression of *Sik3-SA* in PDF neurons increased sleep under DD conditions. **(A)** Average sleep profiles for 30-min intervals of control flies (*Pdf*-*GAL4* × *w^1118^*, light gray line, *n* = 14 and *w^1118^* × *UAS*-*Sik3-SA*, dark gray line, *n* = 10) and flies overexpressing *Sik3-SA* in PDF neurons (*Pdf*-*GAL4* × *UAS*-*Sik3-SA*, orange line, *n* = 20). Day and night under LD and subjective day and night under DD are depicted by white, black, gray, and black bars, respectively. **(B)** Total daily sleep of *Pdf*-*GAL4* × *w^1118^* (light gray bar), *w^1118^* × *UAS*-*Sik3-SA* (dark gray bar) and *Pdf*-*GAL4* × *UAS*-*Sik3-SA* (orange bar) flies under LD and DD conditions (left), and during subjective daytime and nighttime under DD condition (right). **(C)** Average active counts profiles for 30-min intervals of control flies (*Pdf*-*GAL4* × *w^1118^*, gray line, *n* = 14 and *w^1118^* × *UAS*-*Sik3-SA,* dark gray line, *n* = 10) and flies overexpressing *Sik3-SA* in PDF neurons (*Pdf*-*GAL4* × *UAS*-*Sik3-SA*, orange line, *n* = 20). **(D)** Total activity counts of *Pdf*-*GAL4* × *w^1118^* (light gray bar), *w^1118^* × *UAS*-*Sik3-SA* (dark gray bar) and *Pdf*-*GAL4* × *UAS*-*Sik3-SA* (orange bar) flies under LD and DD condition (left), and during subjective daytime and nighttime under DD condition (right). **(E)** Waking activity index of *Pdf*-*GAL4* × *w^1118^* (light gray bar), *w^1118^* × *UAS*-*Sik3-SA* (dark gray bar) and *Pdf*-*GAL4* × *UAS*-*Sik3-SA* (orange bar) flies under LD and DD. **(F)** The period length of *Pdf*-*GAL4* × *w^1118^* (light gray bar, *n* = 14), *w^1118^* × *UAS*-*Sik3-SA* (dark gray bar, *n* = 9) and *Pdf*-*GAL4* × *UAS*-*Sik3-SA* (orange bar, *n* = 9). **(G)** The circadian period amplitude of *Pdf*-*GAL4* × *w^1118^* (light gray bar, *n* = 14), *w^1118^* × *UAS*-*Sik3-SA* (dark gray bar, *n* = 11) and *Pdf*-*GAL4* × *UAS*-*Sik3-SA* (orange bar, *n* = 20). Data are presented as mean ± SEM. #*p* < 0.05 vs. *Pdf*-*GAL4* × *w^1118^*, §:*p* < 0.05 vs. *w^1118^* × *UAS*-*Sik3-SA*; Kruskal-Wallis test followed by Dunn-Bonferroni pairwise comparisons.

**Figure 3:**
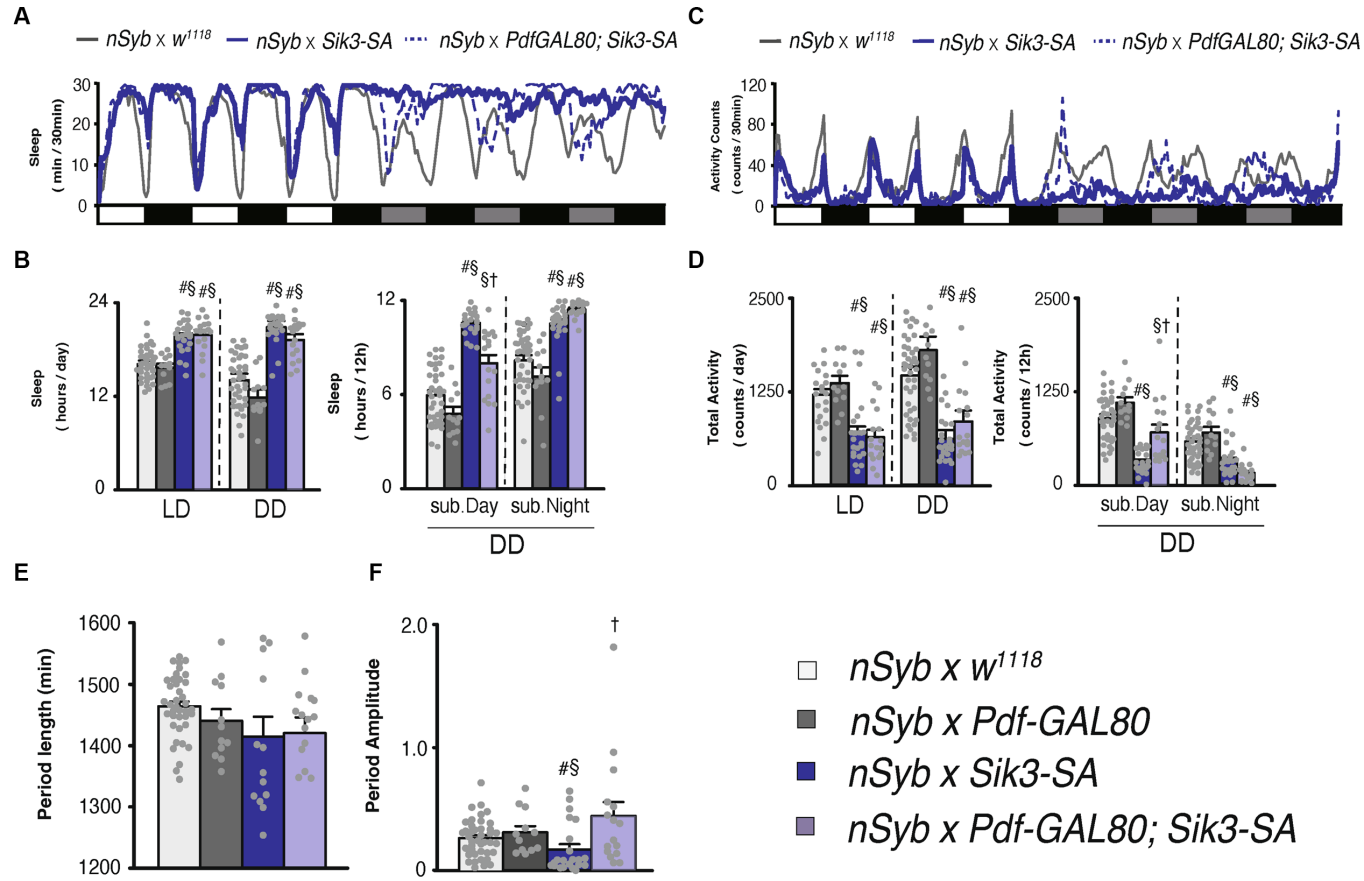
*Pdf*-*GAL80* inhibited the sleep increase caused by *Sik3-SA* overexpression in all neurons specifically during subjective daytime under DD conditions. **(A)** Average sleep profiles for 30-min intervals of control flies (*nSyb*-*GAL4* × *w^1118^*, gray line, *n* = 41) and flies overexpressing *Sik3-SA* in all neurons (*nSyb*-*GAL4* × *UAS*-*Sik3-SA*, blue line, *n* = 25) or in all except *Pdf* neurons (*nSyb*-*GAL4* × *Pdf*-*GAL80*; *UAS*-*Sik3-SA,* blue dotted line, *n* = 16). Day and night under LD and subjective day and night under DD are depicted by white, black, gray, and black bars, respectively. **(B)** Total daily sleep of *nSyb-GAL4* × *w^1118^* (light gray bar), *nSyb-GAL4* × *Pdf-GAL80* (dark gray bar), *nSyb-GAL4* × *UAS*-*Sik3-SA* (blue bar), *nSybGAL4* × *Pdf-GAL80*; *UAS*-*Sik3-SA* (light blue bar) flies under LD and DD condition (left) and during subjective day and night (right). **(C)** Average active counts profiles for 30-min intervals of control flies (*nSyb*-*GAL4* × *w^1118^*, gray line, *n* = 41) and flies overexpressing *Sik3-SA* in all neurons (*nSyb*-*GAL4* × *UAS*-*Sik3-SA*, blue line, *n* = 25) or in all neurons except *Pdf* neurons (*nSyb*-*GAL4* × *Pdf*-*GAL80*; *UAS*-*Sik3-SA,* blue dotted line, *n* = 16). **(D)** Total activity counts of *nSyb-GAL4* x *w^1118^* (light gray bar), *nSyb-GAL4* × *Pdf-GAL80* (dark gray bar), *nSyb-GAL4* × *UAS*-*Sik3-SA* (blue bar), *nSyb-GAL4* × *Pdf*-*GAL80*; *UAS*-*Sik3-SA* (light blue bar) flies under LD and DD condition (left) and during subjective daytime and nighttime (right). **(E)** The period length of *nSyb-GAL4* × *w^1118^* (light gray bar, *n* = 39), *nSyb-GAL4* × *Pdf-GAL80* (dark gray bar, *n* = 12), *nSybGAL4* × *UAS*-*Sik3-SA* (dark blue bar, *n* = 14) and *nSybGAL4* × *Pdf-GAL80*; *UAS*-*Sik3-SA* (light blue bar, *n* = 15). **(F)** The circadian period amplitude of *nSyb-GAL4* × *w^1118^* (light gray bar, *n* = 41), *nSyb-GAL4* × *Pdf-GAL80* (dark gray bar, *n* = 12), *nSyb-GAL4* × *UAS*-*Sik3-SA* (blue bar, *n* = 25), *nSybGAL4* × *Pdf-GAL80*; *UAS*-*Sik3-SA* (light blue bar, *n* = 15). Data are presented as mean ± SEM. #*p* < 0.05 vs. *nSyb*-*GAL4* × *w^1118^*, §:*p* < 0.05 vs. *nSyb-GAL4* × *Pdf-GAL80*, †*p* < 0.05 vs. *nSybGAL4* × *UAS*-*Sik3-SA*; Kruskal-Wallis test followed by Dunn-Bonferroni pairwise comparisons.

Flies (*Drosophila melanogaster*) were reared with conventional food, which contained yeast, glucose, cornmeal, wheat germ, and agar, at 24.5°C under a 12-h:12-h light: dark (LD) cycle as described before (Kume et al., 2005). The following stocks were obtained from the Bloomington Stock Center of Indiana University: *nSyb*-*GAL4* (stock numbers: 68222 and 51,941), *elav*-*GAL4* (458), different *Pdf-GAL4* lines (80,939, 41,286, and 6,899), *R23E10-GAL4* (49032), *elav*-*GS* (43642), *Pdf-GS* (81116) and *UAS-Sik3* RNAi (56941). As control *w^1118^* lines, we used 60,000 (from Bloomington Stock Center, Figures 1, 2) and 2202u (Lab stock derived from CS10, Cantonized *w^1118^*, Figure 3). *Pdf-GAL4* mainly used was provided by Dr. F. R. Jackson, *c232-GAL4* by Dr. J. D. Armstrong, *Pdf*-*GAL80* by Dr. T. Sakai, *DvPdf*-*GAL4* (Bahn et al., 2009) by Dr. T. Yoshii, and *UAS*-*Sik3-SA* transgenic flies were gifts from M. Montminy and J. B. Thomas. We also newly generated AttP site specific transgenic lines for this study. First, we made *UAS-Sik3-WT* (wild type) and *UAS-Sik3-S563A* cDNA by PCR using *pUAST-SIK3 WT* (Addgene Plasmid #52972) and *pUAST-SIK3 S563A* (Addgene Plasmid #52976) as a template, respectively, with the following primers: Forward: 5-GCGGCTCGAG GGTACCAACT TAAAAAAAAA TCAAAATGGC CACCACACCA ACGGGC-3, Reverse: 5-AACGATTCAT TCTAGATTAT AATATTTTAG TTAGCC-3. These cDNAs were introduced by In-fusion (Clontech, USA) into *pJFRC81-10XUAS-IVS-Syn21-GFP-p10* vector (Addgene Plasmid #36432), which was linearized by KpnI-XbaI digestion. Selected clones were validated by DNA sequencing of the inserted DNA. Then transgenic flies were created using *w^1118^* flies with su(Hw)attp5 on chromosome 2 according to standard microinjection methods (Genetic Services, USA).

### Sleep measurement

Male flies, 2- to 5-day-old, were individually housed in glass tubes (length, 65 mm; inner diameter, 3 mm) containing standard fly food at one end and a cotton plug at the other end. For the Gene Switch (GS) system assay and multibeam activity monitor, sucrose-agar food (1% agar supplemented with 5% sucrose) was used instead of standard food. The glass tubes were placed in either the *Drosophila* activity monitor (DAM2) or multibeam activity monitor (MB5) from Trikinetics (MA, USA). In the DAM2, the number of infrared beam crossings was recorded every 1 min, while in the MB5, the number of beam crossings at all 17 positions and the position of the fly in the tube were recorded every 10 s. Sleep was defined as a period of inactivity lasting 5 min or longer. Sleep assays were conducted for 3 days under 12-h light:12-h dark cycle conditions, followed by constant dark (DD) conditions. For conditional expression analysis using GS system, we induced expression by administering the steroid hormone antagonist RU486. Flies were monitored for 2 days in tubes without the drug (pre-food) under DD conditions and then transferred to either the vehicle (0.1% EtOH) or 0.1 mM RU486.

### Sleep deprivation

Flies were individually placed in glass tubes and set in the DAM2 monitor as described above. The DAM2 monitors were then placed onto an Analog MultiTube Vortexer (Fischer Scientific, MA, USA) with a Vortexer Mounting Plate (Trikinetics, MA, USA). Mechanical stimuli, consisting of approximately 1,000 rpm for 10 s, were delivered at 5-min intervals for 12 h during the night to induce sleep deprivation. Locomotor activity was recorded every 1 min. Sleep was calculated for each 6-h period before (pre) and after (post) the mechanical stimulation, as well as for the 12-h period during the mechanical stimulation.

### Circadian behavior analysis

The period length and amplitude of the circadian rhythm were determined by analyzing locomotor activity data collected over 5 days under constant darkness (DD) conditions. Fast Fourier transform (FFT) analysis was performed using a custom-made R code to calculate the power spectrum. Flies with a maximal power spectrum value less than 0.04 or with a peak value outside the range of 1,200 min to 1,680 min were classified as arrhythmic.

### Statistical analysis

Data were analyzed as described in the figure legend, using Microsoft Excel and the freely available statistical software package R 3.5.3 (https://www.r-project.org/).

## Results

### Pan-neuronal *Sik3-SA* overexpression increased sleep robustly under DD condition

Our previous findings demonstrated that conditional adult-specific overexpression of the *Sik3-SA* gene, in which the 563rd serine (equivalent to 551st serine in mice) was replaced with an unphosphorylatable alanine, resulted in a significant increase in sleep (Funato et al., 2016). To validate this phenotype using a constitutive assay, we utilized pan-neuronal *GAL4* drivers, namely *nSyb*-*GAL4* (Figure 1) and *elav*-*GAL4* (Supplementary Figure S1). Sleep measurements were conducted under 12-h light (zeitgeber time, ZT 0–12) followed by 12-h dark (ZT 12–24) cycles, referred to as LD conditions, as well as constant darkness, referred as DD conditions. Constitutive pan-neuronal *Sik3-SA* overexpression also led to an increase in sleep and a decrease in locomotor activity (Figures 1A,C; Supplementary Figures S1A,C). Quantitative analysis of sleep duration and activity levels separately under LD and DD conditions revealed that flies overexpressing *Sik3-SA* exhibited increased total daily sleep and decreased total activity in both LD and DD conditions (Figures 1B,D). A time-course analysis using two-way ANOVA revealed a significant increase in sleep during specific time intervals in the LD condition, as well as in the DD condition (Supplementary Table S1). Notably, the sleep increase induced by *Sik3-SA* overexpression was more prominent in the first half of the 12-h DD period (subjective daytime) compared to the latter half (subjective nighttime) (Figure 1B). The waking activity index, which reflects the average beam crossings (locomotor activity) during wake time, of flies overexpressing *Sik3-SA* in all neurons was slightly higher than that of one control line (*nSyb*-*GAL4* × *w^1118^*) and equivalent to the other control line (*w^1118^* × *UAS*-*Sik3-SA* flies, dark gray bars). To gain more detailed insights into their locomotor activity, we utilized the multibeam activity monitor (MB5) system, which uses 17 infrared beams. Overexpression of *Sik3-SA* in all neurons resulted in a pronounced increase in sleep and a decrease in activity counts under DD conditions (Supplementary Figure S2). Furthermore, we confirmed that flies overexpressing *Sik3-SA* in all neurons could be aroused by light mechanical stimuli when they were in a sleep state (data not shown).

To further investigate the nature of *Sik3-SA* overexpression, we performed sleep deprivation experiments (Supplementary Figures S3A–C). Flies overexpressing *Sik3-SA* in all neurons exhibited a decrease in sleep following mechanical stimuli and displayed a rebound increase in sleep after sleep deprivation. These findings suggest that *Sik3-SA* induces an increase in normal sleep without significant abnormalities or deficits in locomotor activity, sensory input, or sleep homeostasis. Interestingly, *Sik3-SA* overexpressing flies exhibited a more pronounced behavioral phenotype under DD conditions compared to LD conditions. They maintained a consistent sleep duration during subjective daytime under DD, while control flies exhibited decreased sleep under DD compared to LD conditions (Figure 1B; Supplementary Figures S3D,E). Behavioral patterns indicated a reduced amplitude of changes in daily locomotor activity under DD conditions (Figure 1B).

Consequently, we analyzed circadian locomotor rhythmicity for 5 days under DD conditions. Most flies overexpressing *Sik3-SA* in all neurons were classified as arrhythmic with a reduced amplitude of rhythmicity, while 100% of control flies exhibited rhythmicity (Figures 1F,G; Supplementary Figure S4). Only 2 out of 14 flies with *Sik3-SA* overexpression displayed rhythmic behavior (Figure 1F). These results suggest that overexpression of *Sik3-SA* dampens the circadian rhythm of locomotor activity under DD condition.

### Overexpression of *Sik3-SA* in PDF neurons induced an increase in sleep under DD condition

To determine the specific region where *Sik3-SA* functions, we employed region-specific GAL4 drivers to selectively overexpress *Sik3-SA* in limited areas (Supplementary Figure S5). We focused on pigment dispersing factor (PDF) neurons, the dorsal layer of the fan-shaped body (dFB), and the ellipsoid body (EB), as these regions have been implicated in circadian rhythm and sleep regulation. Among them, only the *PDF-GAL4* driver displayed effectiveness under constant dark (DD) conditions (Supplementary Figure S5B). As depicted in Figure 2, overexpression of *Sik3-SA* using *Pdf-GAL4* resulted in significant increases in sleep duration under DD conditions. Similar to pan-neuronal overexpression, it induced alterations in daily sleep patterns specifically during DD conditions. Quantitative analysis revealed a significant increase in sleep and a decrease in activity during the subjective daytime period (Figures 2B,D).

To confirm that this phenotype was indeed caused by PDF neurons, we examined other *GAL4* drivers (BDSC 80939, 41286, and 6899) and *DvPdf-GAL4* that express *GAL4* in *Pdf* (+) cells. All of these drivers exhibited similar effects to PDF-GAL4 (Supplementary Figure S6).

Consistent with the results of pan-neuronal overexpression (Supplementary Figures S3D,E), the relative sleep increase was particularly prominent during the subjective day, corresponding to changes in light conditions (Supplementary Figure S7). Control flies displayed a decrease in sleep during subjective daytime under DD compared to daytime under LD conditions. However, flies overexpressing *Sik3-SA* in PDF neurons exhibited a slight increase in sleep under DD.

We also conducted an analysis of circadian rhythmicity and observed that approximately half of the flies overexpressing *Sik3-SA* in PDF neurons became arrhythmic. No significant differences in the period length were detected (Figures 2F,G; Supplementary Figure S8). These findings suggest that *Sik3-SA* overexpression in PDF neurons disrupts circadian regulation and consequently leads to an increase in sleep during the subjective daytime under DD conditions.

### *Pdf-GAL80* suppressed the sleep increase by pan-neuronal *Sik3-SA* overexpression during the subjective day under DD conditions

Subsequently, we assessed the sleep patterns of flies overexpressing *Sik3-SA* in all neurons except for PDF neurons using *Pdf-GAL80*. As expected, pan-neuronal *Sik3-SA* overexpression driven by *nSyb-GAL4* resulted in an increase in sleep duration and a decrease in locomotor activity, confirming the findings from Figure 1. Then, by employing *Pdf-GAL80*, we observed that the effects of *Sik3-SA* on sleep were specifically suppressed during the subjective daytime under DD conditions (Figures 3A–D). This outcome aligns with the effects of *Sik3-SA* overexpression in PDF neurons, which induced increased sleep during the subjective daytime. Additionally, expression of *Pdf-GAL80* in PDF neurons restored the amplitude of the circadian rhythm, with approximately 94% of the flies classified as rhythmic (Supplementary Figure S9). Collectively, these results indicate that the overexpression of *Sik3-SA* in PDF neurons plays a dominant role in promoting increased sleep and destabilizing circadian rhythmicity under DD conditions.

### Adult-specific conditional overexpression of *Sik3-SA* in PDF neurons increased sleep

**Figure 4:**
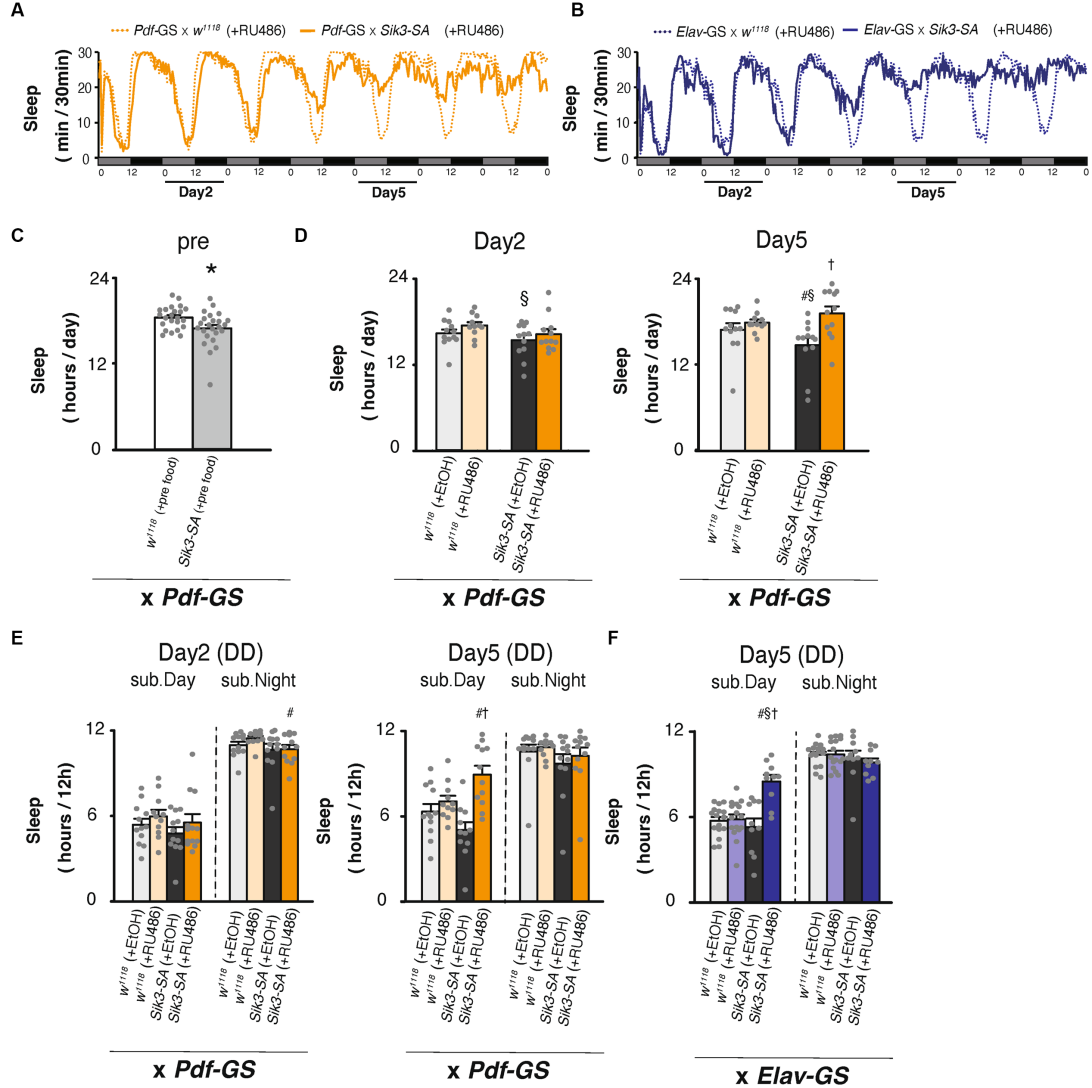
Conditional overexpression of *Sik3-SA* specifically in PDF neurons in adult flies resulted in an increase in sleep. **(A)** Average sleep profiles for 30-min intervals of control flies (*Pdf*-GS × *w^1118^* [+RU486], orange dotted lines, *n* = 12) and flies with conditional overexpression of *Sik3-SA* in PDF neurons (*Pdf*-GS × *UAS*-*Sik3-SA* [+RU486], orange lines, *n* = 12). **(B)** Average sleep profiles for 30-min intervals of control flies (*elav*-GS × *w^1118^* [+RU486], blue dotted lines, *n* = 16) and flies with conditional overexpression of *Sik3-SA* in all neurons (*elav-*GS × *UAS*-*Sik3-SA* [+RU486], blue lines, *n* = 10). Each fly was recorded for 7 days under DD conditions for 2 days in 5% suc and 1% agar (pre-food). Subjective days and nights are indicated by gray and black bars, respectively. **(C)** Total daily sleep of control flies (*Pdf*-GS × *w^1118^* [pre-food], white bar, *n* = 23) and flies with conditional overexpression of *Sik3-SA* in PDF neurons (*Pdf*-GS × *UAS*-*Sik3-SA* [pre-food], gray bar, *n* = 24) in the pre-period. **(D)** Total daily sleep of control flies (*Pdf*-GS × *w^1118^* [+EtOH], pale gray bar, *n* = 12; *Pdf*-GS × *w^1118^* [*+*RU486], pale orange bar, *n* = 11; and *Pdf*-GS × *UAS*-*Sik3-SA* [+EtOH], dark gray bar, *n* = 12) and flies with conditional overexpression of *Sik3-SA* in PDF neurons (*Pdf*-GS × *UAS*-*Sik3-SA* [+RU486], dark orange bar, *n* = 12) on day 2 and day 5. **(E)** Total daily sleep of control flies (*Pdf*-GS × *w^1118^* [+EtOH], pale gray bar; *Pdf*-GS × *w^1118^* [*+*RU486], pale orange bar; and *Pdf*-GS × *UAS*-*Sik3-SA* [+EtOH], dark gray bar) and flies with the conditional overexpression of *Sik3-SA* in PDF neurons (*Pdf*-GS × *UAS*-*Sik3-SA* [+RU486], dark orange bar) during the subjective day and night on day 2 and day 5. **(F)** Total daily sleep of control flies (*elav-*GS × *w^1118^* [+EtOH], pale gray bar, *n* = 16; *elav*-GS × *w^1118^* [*+*RU486], pale blue bar, *n* = 10; and *elav*-GS × *UAS*-*Sik3-SA* [+EtOH], dark gray bar, *n* = 16) and flies with conditional overexpression of *Sik3-SA* in all neurons (*elav*-GS × *UAS*-*Sik3-SA* [+RU486], dark blue bar, *n* = 10) during the subjective day and night on day 5. Data are presented as the mean ± SEM. #*p* < 0.05 vs. × *w^1118^* [+EtOH], §:*p* < 0.05, vs. × *w*^*111*8^ [+RU486], †*p* < 0.05 vs. × *UAS*-*Sik3-SA* [+EtOH]; Tukey–Kramer method. ^*^p < 0.05; Welch’s *t*-test.

To exclude developmental effects, we utilized the Gene Switch (GS) system to selectively overexpress *Sik3-SA* in PDF neurons of adult flies (Osterwalder et al., 2001; Roman et al., 2001). Flies expressing GS in PDF neurons, with or without *UAS-Sik3-SA*, were provided with a diet of 5% sucrose and 1% agar (pre-food) for 2 days in glass tubes. Subsequently, they were transferred to new tubes containing either RU486 (100 μM) or the vehicle, EtOH, at circadian time (CT) 0. Following the food change, flies with *Sik3-SA* overexpression in PDF neurons gradually exhibited increased sleep compared to control flies, resembling the changes observed in flies with pan-neuronal *Sik3-SA* overexpression (Figures 4A,B). During both the pre-food period and day 2 with RU486, all groups displayed a similar amount of sleep (Figures 4C,D). On day 5, no significant difference in sleep was observed between control flies [*Pdf*-*GS* ×*w^1118^*] treated with EtOH and those treated with RU486. However, flies overexpressing *Sik3-SA* through RU486 treatment [*Pdf*-*GS* × *UAS-Sik3-SA* (+ RU486)] exhibited increased sleep compared to the EtOH-treated groups [*Pdf*-*GS* × *UAS-Sik3-SA* (+ EtOH)] (Figure 4D). The effect on sleep duration appeared to be more pronounced during the subjective daytime than the nighttime on Day 5, but not on Day 2 (Figure 4E). To investigate the disparity in sleep patterns between LD and DD conditions, we conducted a similar experiment under LD conditions and transferred the flies from LD to DD between days 6 and 7 (Supplementary Figure S10A, black arrow). Although the period during which the flies were administered RU486 remained the same as in Figure 4, there was no change in sleep duration on day 5 under LD conditions (Supplementary Figure S10B). However, upon switching from LD to DD conditions, flies with induced overexpression of *Sik3-SA* through RU486 treatment immediately exhibited a noticeable increase in sleep during the subjective daytime (Supplementary Figures S10C,D). These results indicate that the changes in sleep induced by overexpression of *Sik3-SA* in PDF neurons are latent and not evident under LD conditions, but become prominent under DD conditions, particularly during the subjective daytime period.

To quantitatively compare wild type *Sik3* and *Sik3-SA*, we aimed to achieve the same expression level for both constructs. Therefore, we generated new transgenic lines, *UAS-Sik3-WT* and *UAS-Sik3-SA*, using the AttP-based site-specific integration method. However, when we crossed these lines with pan-neuronal GAL4 drivers such as *elav-GAL4* or *nSyb- GAL4*, no viable offspring were obtained. As an alternative, we employed the GS system and crossed the *UAS-Sik3-WT* and *UAS-Sik3-SA* lines with *PDF-GS* flies. Intriguingly, we found that only *Sik3-SA*, but not *Sik3-WT*, increased sleep following RU486 administration (Supplementary Figure S11).

### *Sik3* knock-down by RNAi in PDF neurons lengthened the circadian periodicity

**Figure 5:**
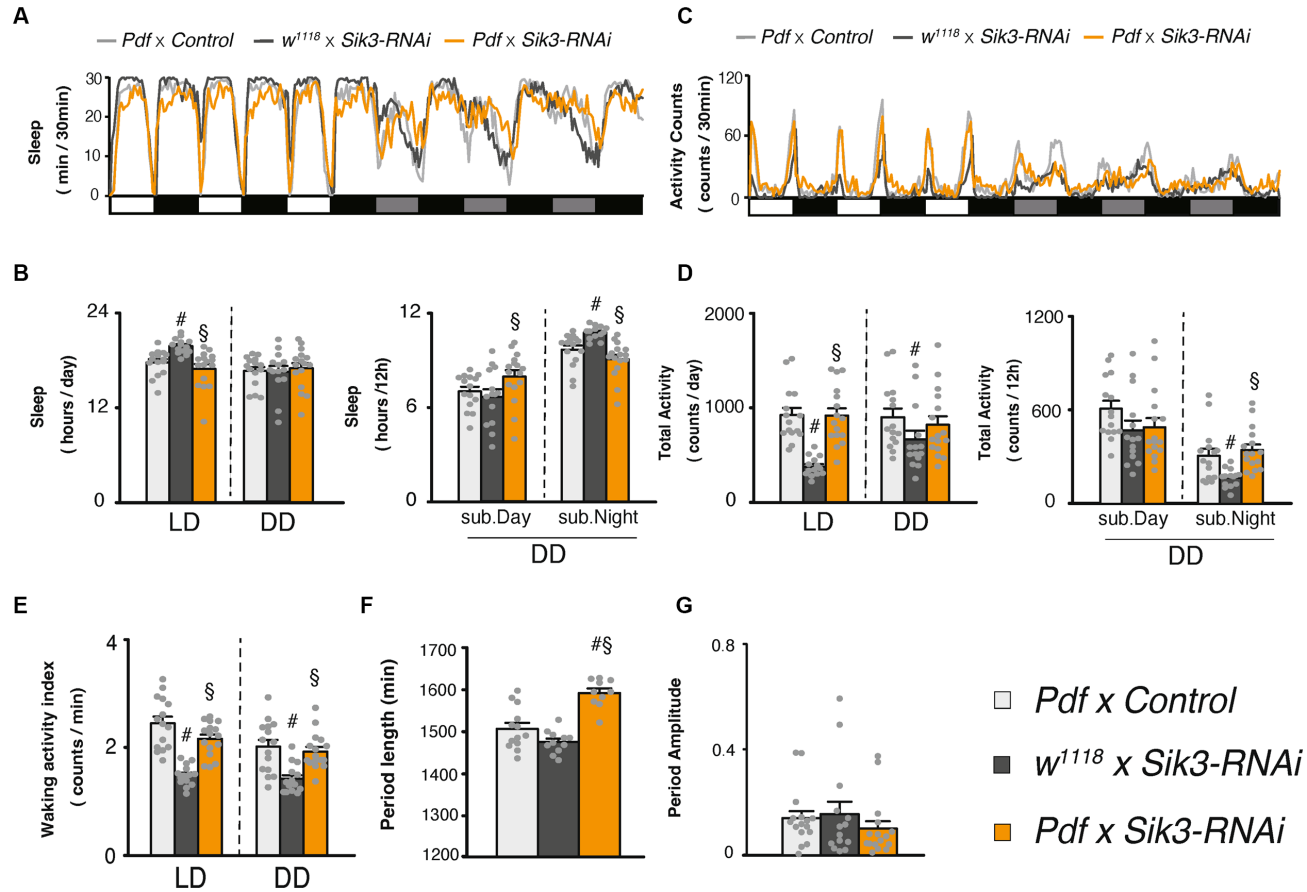
Knockdown of *Sik3* specifically in PDF neurons led to a lengthening of the circadian rhythm. **(A)** Average sleep profiles for 30-min intervals of control flies (*Pdf*-*GAL4* × control [BDSC 36303], light gray line, *n* = 15 and *w^1118^* × *UAS*-*Sik3-RNAi*, dark gray line, *n* = 14) and flies with knockdown of *Sik3* in PDF neurons (*Pdf*-*GAL4* × *UAS*-*Sik3-RNAi*, orange line, *n* = 16). Day and night under LD and subjective day and night under DD are depicted by white, black, gray, and black bars, respectively. **(B)** Total daily sleep of *Pdf*-*GAL4* × control [BDSC 36303] (light gray bar), *w^1118^* × *UAS*-*Sik3-RNAi* (dark gray bar) and *Pdf*-*GAL4* × *UAS*-*Sik3-RNAi* (orange bar) flies under LD and DD (left) and during subjective daytime and nighttime under DD condition (right). **(C)** Average active counts profiles for 30-min intervals of control flies (*Pdf*-*GAL4* × control [BDSC 36303], light gray line, *n* = 15 and *w^1118^* × *UAS*-*Sik3-RNAi*, dark gray line, *n* = 14) and flies with knockdown of *Sik3* in PDF neurons (*Pdf*-*GAL4* × *UAS*-*Sik3-RNAi*, orange line, *n* = 16). **(D)** Total activity counts of *Pdf*-*GAL4* × control [BDSC 36303] (light gray bar), *w^1118^* × *UAS*-*Sik3-RNAi* (dark gray bar) and *Pdf*-*GAL4* × *UAS*-*Sik3-RNAi* (orange bar) flies under LD and DD condition (left) and during subjective day (DD 0-12) and night (DD 12-24) under DD condition (right). **(E)** Waking activity index of *Pdf*-*GAL4* × control [BDSC 36303] (light gray bar), *w^1118^* × *UAS*-*Sik3-RNAi* (dark gray bar) and *Pdf*-*GAL4* × *UAS*-*Sik3-RNAi* (orange bar) flies under LD and DD. **(F)** The period length of *Pdf*-*GAL4* × control [BDSC 36303] (light gray bar, *n* = 15), *w^1118^* × *UAS*-*Sik3-RNAi* (dark gray bar, *n* = 14) and *Pdf*-*GAL4* × *UAS*-*Sik3-RNAi* (orange bar, *n* = 16) flies. **(G)** The circadian period amplitude of *Pdf*-*GAL4* × control [BDSC 36303] (light gray bar, *n* = 15), *w^1118^* × *UAS*-*Sik3-RNAi* (dark gray bar, *n* = 14) and *Pdf*-*GAL4* × *UAS*-*Sik3-RNAi* (orange bar, *n* = 16) flies. Data are presented as mean ± SEM; # *p* < 0.05 vs. *Pdf*-*GAL4* × control, §:*p* < 0.05 vs. *w^1118^* × *UAS*-*Sik3-RNAi*; Kruskal-Wallis test followed by Dunn-Bonferroni pairwise comparisons.

Since *Sik3-SA* overexpression in PDF neurons led to a reduction in the amplitude of the circadian rhythm, we investigated the effects of *Sik3* knock-down specifically in PDF neurons using RNAi. However, the results were not consistent across two control group. One control line, *w^1118^* × *UAS-Sik3 RNAi* showed a significant increase in sleep duration and a decrease in the waking activity index compared with the other control line, *Pdf-Gal4* × *w^1118^*. In contrast, *Sik3* knock-down did not appear to have a notable impact on sleep duration (Figures 5A,B,D). On the other hand, we did observe a significant lengthening of circadian periods upon *Sik3* knock-down compared to the two control groups (Figure 5F).

## Discussion

In this study, we demonstrated that the overexpression of the *Sik3-SA* mutant gene, which lacks phosphorylation at serine 563, in all neurons led to a decrease in total locomotor activity counts and an increase in sleep duration under both LD and DD conditions in *Drosophila* (Figure 1). Additionally, we observed a reduction in the circadian amplitude of sleep and activity rhythms specifically under DD conditions. Notably, the overexpression of *Sik3-SA* in all neurons did not significantly affect the waking activity index (Figure 1; Supplementary Figure S2C) or the response to mechanical stimulation (Supplementary Figures S3A–C). Moreover, the rebound sleep observed after sleep deprivation in flies with pan-neuronal *Sik3-SA* overexpression was comparable to that of control flies (Supplementary Figure S3C). These findings suggest that the overexpression of *Sik3-SA* primarily modulates sleep duration (reducing wakefulness) without causing abnormalities in locomotor activity, sensory input, or sleep homeostasis. These results are consistent with previous findings in *Sleepy* mice, which exhibit significantly longer sleep duration compared to wild-type mice while maintaining overall health (Funato et al., 2016; Honda et al., 2018; Wang et al., 2018).

We subsequently observed that selective overexpression of the *Sik3-SA* mutant gene in PDF neurons resulted in an increase in sleep duration, particularly during subjective daytime under DD conditions, accompanied by a significant reduction in the amplitude of circadian activity rhythms (Figure 2). Notably, overexpression of *Sik3-SA* in other regions, including dFB and EB, did not elicit significant changes in sleep (Supplementary Figure S5). Moreover, adult-specific overexpression of *Sik3-SA* in PDF neurons also induced sleep elevation during subjective daytime under DD conditions (Figure 4), suggesting that PDF neurons specifically regulate sleep in adult flies. We further confirmed the sleep-promoting effect of *Sik3-SA* overexpression in PDF neurons using different GAL4 drivers that express GAL4 in PDF neurons (Supplementary Figure S6). Interestingly, some of these PDF-GAL4 drivers also exhibited increased sleep duration under LD conditions. This observation suggests the possibility of the expression in additional regions besides PDF neurons or stronger expression levels in PDF neurons by these GAL4 drivers. Further investigation is required to determine the cause for this difference. Nonetheless, since the expression of GAL80 in PDF neurons in flies with pan-neuronal *Sik3-SA* overexpression attenuated the sleep-promoting phenotype under DD conditions but had a less pronounced effect under LD conditions (Figure 3), we concluded that PDF neurons play a central role in mediating the sleep phenotype induced by *Sik3-SA* specifically under DD conditions.

To explore the distinction between wild-type *Sik3* and *Sik3-SA*, we utilized the same *UAS* transgene construct to ensure comparable expression levels of the transgenes. Notably, overexpression of *Sik3-SA* specifically in PDF neurons resulted in an increase in sleep, whereas overexpression of wild-type *Sik3* did not produce a similar effect (Supplementary Figure S11). This finding highlights the differential nature and functional consequences of *Sik3-SA* compared to wild-type *Sik3*.

Next, we performed knockdown experiments targeting *Sik3* specifically in PDF neurons, which resulted in a lengthening of the circadian period without significant effects on sleep amount (Figure 5). A previous study demonstrated that complete deletion of *Sik3* in *Drosophila* was lethal, but restoring its expression in the fat body rescued this lethality, albeit with resulting arrhythmicity in locomotor activity (Fujii et al., 2017). However, when *Sik3* expression was restored in both the fat body and PDF neurons, the circadian rhythm of locomotor activity was rescued. Although the sleep phenotype was not addressed in that study, their findings emphasized the importance of *Sik3* in PDF neurons for regulating circadian rhythm. Furthermore, they reported that *Sik3* knockdown in PDF neurons did not impact the rhythmicity of the PERIOD protein. Collectively, these results suggest that *Sik3* may play a physiological role in regulating circadian rhythm specifically in PDF neurons. Although we did not analyze the oscillation of clock genes or PDF at the protein level in clock neurons, our results and previous results from other studies suggest that *Sik3-SA* may primarily influence the circadian output of PDF neurons, rather than directly impacting their core clock oscillation. Further investigations are required to fully understand the underlying mechanisms.

PDF neurons are well-known regulators of the circadian system and they play a crucial role in maintaining the activity of both clock and non-clock neurons through the secretion of the PDF peptide. In the absence of PDF signaling, flies gradually lose their circadian rhythmicity under DD conditions (Renn et al., 1999). While the sleep regulation by PDF neurons has been extensively studied under LD conditions, only a few studies have investigated their role in sleep regulation under DD conditions (Chung et al., 2009; Klose and Shaw, 2021). Previous studies have shown that flies with ablated PDF neurons (through *UAS-hid* expression), PDF null mutants (*Pdf^01^*), or PDF receptor null mutants (*PDFRhan^5304^*) exhibited increased sleep under DD conditions, with more pronounced effects observed during the subjective day compared to the subjective night. These findings are consistent with our results, indicating that PDF neurons are involved in sleep regulation under DD conditions.

PDF neurons play a crucial role in regulating the sleep/wake cycle through various pathways. (1) In response to light, the activation of l-LNv and s-LNv neurons leads to the release of the PDF peptide. (2) The PDF receptor (PDFR) present in PDFR-expressing neurons is activated by PDF, thereby regulating the sleep/wake state. Within the clock neurons, DN1p neurons are activated by the PDF peptide and transmit waking signals to both wake-promoting neurons (such as *Dilp2*-expressing cells) and the ellipsoid body (EB), which is involved in motor control through TuBu neurons (Guo et al., 2016, 2018). In the EB, the interaction between PDF and PDFR leads to elevated cAMP levels and increased activity. Thus, the secretion of PDF peptides in response to light triggers wakefulness (Parisky et al., 2008; Shang et al., 2008). Additionally, it has been suggested that sleep-promoting PPM3 neurons express PDFRs and exhibit changes in their expression levels in response to daytime sleep. It has been proposed that PDF neurons establish direct synaptic connections with sleep-promoting PPM3 neurons and inhibit these connections (Potdar and Sheeba, 2018; Liang et al., 2019). (3) During nighttime, GABAergic sleep-promoting neurons inhibit both LNvs through the GABA_A_ receptor (*Rdl*), while glutamatergic neurons in DN1s release glutamate to inhibit wake signals in s-LNv neurons (Agosto et al., 2008; Parisky et al., 2008). However, it remains unknown whether these pathways also operate under DD conditions.

In murine experiments, the homozygous knockout of *Sik3* resulted in lethality, and the surviving homozygotes exhibited alterations in circadian rhythm and reduced size and strength (Hayasaka et al., 2017). Similarly, in flies, *Sik3* is essential for survival, and homozygous knockout flies are lethal. Our *RNAi* experiment (Figure 5) further supports the physiological role of *Sik3* in the regulation of circadian rhythm. These findings indicate that *Sik3* plays a conserved role in circadian regulation across species.

In mice, the *Sleepy* mutation causes a significant increase in sleep, particularly during the night (dark) period, as mice are nocturnal animals. It is believed that the *Sleepy* mutation acts dominantly, as heterozygous mice display a similar phenotype to homozygous mice (Funato et al., 2016). The *Sleepy* mutation is thought to increase sleepiness, resembling the effects of sleep deprivation. It has been proposed that the proteins whose phosphorylation is enhanced in both *Sleepy* mutant mice and sleep-deprived mice represent a measure of sleep need, termed sleep need index phosphoproteins (SNIPPs) (Wang et al., 2018). Interestingly, we have identified a *Drosophila* homolog of one of the SNIPPs genes that is involved in sleep regulation (Kobayashi et al., 2023). Recent studies in mice have shown that SIK3 regulates sleep quantity and depth differently in different brain regionss (Kim et al., 2022). Moreover, the expression of *SIK3-SA* in the suprachiasmatic nucleus, the central pacemaker of circadian rhythm in mammals, altered the daily activity profile (Asano et al., 2023). These findings align well with the results of our study. However, at present, we lack clear mechanistic evidence on how *Sik3-SA* alters the function of normal *Sik3* and the specific mechanism by which *Sik3-SA* modulates the function of PDF neurons remains unclear. The current findings suggest a potential link between *Sik3-SA* and the output of PDF neurons, but additional studies are necessary to confirm and expand upon these observed effects. To further investigate this link, additional experiments, such as assessing the expression and oscillation patterns of PER and PDF proteins specifically in PDF neurons would be valuable. These experiments would provide more insights into the potential interference of *Sik3-SA* with the output from PDF neurons, which may contribute to the observed reduction in circadian rhythmicity and sleep.

## Data availability statement

The raw data supporting the conclusions of this article will be made available by the authors, without undue reservation.

## Author contributions

RK, SN, JT, HF, MY, and KK designed the experiments and wrote the manuscript. RK and SN conducted all experiments and data analysis. All authors contributed to the article and approved the submitted version.

## Funding

This study was supported by JSPS, Japan: Kazuhiko Kume 18H02481 and 21H02529; Jun Tomita 20K06744; Riho Kobayashi 20J23449.

## Conflict of interest

The authors declare that the research was conducted in the absence of any commercial or financial relationships that could be construed as a potential conflict of interest.

## Publisher’s note

All claims expressed in this article are solely those of the authors and do not necessarily represent those of their affiliated organizations, or those of the publisher, the editors and the reviewers. Any product that may be evaluated in this article, or claim that may be made by its manufacturer, is not guaranteed or endorsed by the publisher.

## References

[ref1] Agosto J. Choi J. C. Parisky K. M. Stilwell G. Rosbash M. Griffith L. C. (2008). Modulation of GABAA receptor desensitization uncouples sleep onset and maintenance in *Drosophila*. Nat. Neurosci. 11, 354–359. doi: 10.1038/nn2046, PMID: 18223647 PMC2655319

[ref2] Asano F. Kim S. J. Fujiyama T. Miyoshi C. Hotta-Hirashima N. Asama N.. (2023). SIK3–HDAC4 in the suprachiasmatic nucleus regulates the timing of arousal at the dark onset and circadian period in mice. Proc. Natl. Acad. Sci. 120:2017. doi: 10.1073/pnas.2218209120 PMC10089210 36877841

[ref3] Bahn J. H. Lee G. Park J. H. (2009). Comparative analysis of pdf-mediated circadian behaviors between Drosophila melanogaster and *D. virilis*. Genetics 181, 965–975. doi: 10.1534/genetics.108.099069, PMID: 19153257 PMC2651067

[ref4] Borbély A. A. Daan S. Wirz-Justice A. Deboer T. (2016). The two-process model of sleep regulation: a reappraisal. J. Sleep Res. 25, 131–143. doi: 10.1111/jsr.12371, PMID: 26762182

[ref5] Choi S. Lim D. S. Chung J. (2015). Feeding and fasting signals converge on the LKB1-SIK3 pathway to regulate lipid metabolism in *Drosophila*. PLoS Genet. 11, 1–19. doi: 10.1371/journal.pgen.1005263 PMC4440640 25996931

[ref6] Chung B. Y. Kilman V. L. Keath J. R. Pitman J. L. Allada R. (2009). The GABA(A) receptor RDL acts in peptidergic PDF neurons to promote sleep in *Drosophila*. Curr. Biol. 19, 386–390. doi: 10.1016/j.cub.2009.01.040, PMID: 19230663 PMC3209479

[ref7] Fujii S. Emery P. Amrein H. (2017). SIK3-HDAC4 signaling regulates Drosophila circadian male sex drive rhythm via modulating the DN1 clock neurons. Proc. Natl. Acad. Sci. U. S. A. 114, E6669–E6677. doi: 10.1073/pnas.1620483114, PMID: 28743754 PMC5558993

[ref8] Funato H. Miyoshi C. Fujiyama T. Kanda T. Sato M. Wang Z.. (2016). Forward-genetics analysis of sleep in randomly mutagenized mice. Nature 539, 378–383. doi: 10.1038/nature20142, PMID: 27806374 PMC6076225

[ref10] Guo F. Holla M. Díaz M. M. Rosbash M. (2018). A circadian output circuit controls sleep-wake arousal in *Drosophila*. Neuron 100, 624–635.e4. doi: 10.1016/j.neuron.2018.09.002, PMID: 30269992

[ref11] Guo F. Yu J. Jung H. J. Abruzzi K. C. Luo W. Griffith L. C.. (2016). Circadian neuron feedback controls the *Drosophila* sleep-activity profile. Nature 536, 292–297. doi: 10.1038/nature19097, PMID: 27479324 PMC5247284

[ref12] Hasegawa T. Tomita J. Hashimoto R. Ueno T. Kume S. (2017). Sweetness induces sleep through gustatory signalling independent of nutritional value in a starved fruit fly. Sci. Rep. 7. doi: 10.1038/s41598-017-14608-1 PMC5662574 29084998

[ref13] Hayasaka N. Hirano A. Miyoshi Y. Tokuda I. T. Yoshitane H. Matsuda J.. (2017). Salt-inducible kinase 3 regulates the mammalian circadian clock by destabilizing PER2 protein. elife 6, 1–17. doi: 10.7554/eLife.24779 PMC5747517 29227248

[ref14] Hendricks J. C. Finn S. M. Panckeri K. A. Chavkin J. Williams J. A. Sehgal A.. (2000). Rest in *Drosophila* is a sleep-like state. Neuron 25, 129–138. doi: 10.1016/S0896-6273(00)80877-6, PMID: 10707978

[ref15] Honda T. Fujiyama T. Miyoshi C. Ikkyu A. Hotta-Hirashima N. Kanno S.. (2018). A single phosphorylation site of SIK3 regulates daily sleep amounts and sleep need in mice. Proc. Natl. Acad. Sci. U. S. A. 115, 10458–10463. doi: 10.1073/pnas.1810823115, PMID: 30254177 PMC6187192

[ref16] Kim S. J. Hotta-Hirashima N. Asano F. Kitazono T. Iwasaki K. Nakata S.. (2022). Kinase signalling in excitatory neurons regulates sleep quantity and depth. Nature 612, 512–518. doi: 10.1038/s41586-022-05450-1, PMID: 36477539

[ref17] Klose M. K. Shaw P. J. (2021). Sleep drive reconfigures wake-promoting clock circuitry to regulate adaptive behavior. PLoS Biol. 19, 1–22. doi: 10.1371/journal.pbio.3001324 PMC8277072 34191802

[ref18] Kobayashi R. Yamashita Y. Suzuki H. Hatori S. Tomita J. Kume K. (2023). rdgB knockdown in neurons reduced nocturnal sleep in *Drosophila melanogaster*. Biochem. Biophys. Res. Commun. 643, 24–29. doi: 10.1016/j.bbrc.2022.12.043, PMID: 36586155

[ref19] Kume K. Kume S. Park S. K. Hirsh J. Jackson F. R. (2005). Dopamine is a regulator of arousal in the fruit fly. J. Neurosci. 25, 7377–7384. doi: 10.1523/JNEUROSCI.2048-05.2005, PMID: 16093388 PMC6725300

[ref20] Liang X. Ho M. C. W. Zhang Y. Li Y. Wu M. N. Holy T. E.. (2019). Morning and evening circadian pacemakers independently drive premotor centers via a specific dopamine relay. Neuron 102, 843–857.e4. doi: 10.1016/j.neuron.2019.03.028, PMID: 30981533 PMC6533154

[ref21] Nakagawa H. Maehara S. Kume K. Ohta H. Tomita J. (2022a). Biological functions of α2-adrenergic-like octopamine receptor in *Drosophila melanogaster*. Genes Brain Behav. 21:e12807. doi: 10.1111/gbb.12807 35411674 PMC9744561

[ref22] Nakagawa H. Nakane S. Ban G. Tomita J. Kume K. (2022b). Effects of D-amino acids on sleep in *Drosophila*. Biochem. Biophys. Res. Commun. 589, 180–185. doi: 10.1016/j.bbrc.2021.11.107, PMID: 34922200

[ref23] Osterwalder T. Yoon K. S. White B. H. Keshishian H. (2001). A conditional tissue-specific transgene expression system using inducible GAL4. Proc. Natl. Acad. Sci. U. S. A. 98, 12596–12601. doi: 10.1073/pnas.221303298, PMID: 11675495 PMC60099

[ref24] Parisky K. M. Agosto J. Pulver S. R. Shang Y. Kuklin E. Hodge J. J. L.. (2008). PDF cells are a GABA-responsive wake-promoting component of the *Drosophila* sleep circuit. Neuron 60, 672–682. doi: 10.1016/j.neuron.2008.10.042, PMID: 19038223 PMC2734413

[ref26] Potdar S. Sheeba V. (2018). Wakefulness is promoted during day time by PDFR signalling to dopaminergic neurons in *Drosophila melanogaster*. eNeuro 5:ENEURO.0129-18.2018. doi: 10.1523/ENEURO.0129-18.2018 PMC6102377 30131970

[ref27] Renn S. C. Park J. H. Rosbash M. Hall J. C. Taghert P. H. (1999). A *Pdf* neuropeptide gene mutation and ablation of PDF neurons each cause severe abnormalities of behavioral circadian rhythms in *Drosophila*. Cells 99, 791–802. doi: 10.1016/S0092-8674(00)81676-1, PMID: 10619432

[ref28] Roman G. Endo K. Zong L. Davis R. L. (2001). P{switch}, a system for spatial and temporal control of gene expression in *Drosophila melanogaster*. Proc. Natl. Acad. Sci. U. S. A. 98, 12602–12607. doi: 10.1073/pnas.221303998, PMID: 11675496 PMC60100

[ref29] Shang Y. Griffith L. C. Rosbash M. (2008). Light-arousal and circadian photoreception circuits intersect at the large PDF cells of the *Drosophila* brain. Proc. Natl. Acad. Sci. U. S. A. 105, 19587–19594. doi: 10.1073/pnas.0809577105, PMID: 19060186 PMC2596742

[ref30] Shaw P. J. Cirelli C. Greenspan R. J. Tononi G. (2000). Correlates of sleep and waking in *Drosophila melanogaster*. Science 287, 1834–1837. doi: 10.1126/science.287.5459.1834, PMID: 10710313

[ref31] Tomita J. Ban G. Kume K. (2017). Genes and neural circuits for sleep of the fruit fly. Neurosci. Res. 118, 82–91. doi: 10.1016/j.neures.2017.04.010, PMID: 28438481

[ref32] Tomita J. Mitsuyoshi M. Ueno T. Aso Y. Tanimoto H. Nakai Y.. (2011). Pan-neuronal knockdown of calcineurin reduces sleep in the fruit fly, *Drosophila* melanogaster. J. Neurosci. 31, 13137–13146. doi: 10.1523/JNEUROSCI.5860-10.2011 21917797 PMC6623252

[ref33] Ueno T. Tomita J. Tanimoto H. Endo K. Ito K. Kume S.. (2012). Identification of a dopamine pathway that regulates sleep and arousal in *Drosophila*. Nat. Neurosci. 15, 1516–1523. doi: 10.1038/nn.3238, PMID: 23064381

[ref34] Wang Z. Ma J. Miyoshi C. Li Y. Sato M. Ogawa Y.. (2018). Quantitative phosphoproteomic analysis of the molecular substrates of sleep need. Nature 558, 435–439. doi: 10.1038/s41586-018-0218-8, PMID: 29899451 PMC6350790

[ref35] Yamaguchi S. T. Kobayashi R. Tomita J. Kume K. (2022a). The regulation of circadian rhythm by insulin signaling in Drosophila. Neurosci. Res. 183, 76–83. doi: 10.1016/j.neures.2022.07.005, PMID: 35872183

[ref36] Yamaguchi S. T. Tomita J. Kume K. (2022b). Insulin signaling in clock neurons regulates sleep in *Drosophila*. Biochem. Biophys. Res. Commun. 591, 44–49. doi: 10.1016/j.bbrc.2021.12.100, PMID: 34998032

