# Supplementary material for: A phosphorylation-deficient mutant of Sik3, a homolog of Sleepy, alters circadian sleep regulation by PDF neurons in Drosophila
Source: Front Neurosci. 2023 Aug 17;17:1181555. doi: 10.3389/fnins.2023.1181555 (PMC10469759; doi:10.3389/fnins.2023.1181555)
Supplement: Supplementary file 1 [file Data_Sheet_1.PDF]

Table S1. *nSyb x w1118 vs nSyb x Sik3-SA*

| | | | DFn, DFd | F-ration | p-value | | | | | F-ration | p-value |
|---|---|---|---|---|---|---|---|---|---|---|---|
| Fig.1 (A) total sleep | LD1 | ***ZT 0*** | 1,23 | 6.0919 | 0.0214 * | Fig.1 (A) total sleep | DD1 | ***CT 0*** | 1,23 | 53.5733 | < 0.00001 *** |
| | | ***ZT 1*** | 1,23 | 22.7285 | 0.0001 *** | | | ***CT 1*** | 1,23 | 29.1674 | < 0.00001 *** |
| | | ***ZT 2*** | 1,23 | 11.5111 | 0.0025 ** | | | ***CT 2*** | 1,23 | 7.0748 | 0.0140 * |
| | | ZT 3 | 1,23 | 0.0531 | 0.8198 ns | | | CT 3 | 1,23 | 1.3765 | 0.2527 ns |
| | | ZT 4 | 1,23 | 0.715 | 0.4065 ns | | | CT 4 | 1,23 | 0.4813 | 0.4948 ns |
| | | ZT 5 | 1,23 | 1.4688 | 0.2378 ns | | | CT 5 | 1,23 | 0.1104 | 0.7427 ns |
| | | ZT 6 | 1,23 | 0.5672 | 0.4590 ns | | | CT 6 | 1,23 | 0.3126 | 0.5815 ns |
| | | ZT 7 | 1,23 | 1.2758 | 0.2703 ns | | | ***CT 7*** | 1,23 | 4.5379 | 0.0441 * |
| | | ZT 8 | 1,23 | 0.4456 | 0.5111 ns | | | ***CT 8*** | 1,23 | 9.8564 | 0.0046 ** |
| | | ZT 9 | 1,23 | 0.1326 | 0.7191 ns | | | ***CT 9*** | 1,23 | 12.8305 | 0.0016 ** |
| | | ZT 10 | 1,23 | 1.0938 | 0.3065 ns | | | ***CT 10*** | 1,23 | 38.0116 | < 0.00001 *** |
| | | ***ZT 11*** | 1,23 | 14.9374 | 0.0008 *** | | | ***CT 11*** | 1,23 | 43.9233 | < 0.00001 *** |
| | | ***ZT 12*** | 1,23 | 16.8341 | 0.0004 *** | | | ***CT 12*** | 1,23 | 77.875 | < 0.00001 *** |
| | | ***ZT 13*** | 1,23 | 4.5218 | 0.0444 * | | | ***CT 13*** | 1,23 | 56.0032 | < 0.00001 *** |
| | | ***ZT 14*** | 1,23 | 0.074 | 0.7880 ns | | | ***CT 14*** | 1,23 | 11.1304 | 0.0029 ** |
| | | ZT 15 | 1,23 | 0.2578 | 0.6164 ns | | | CT 15 | 1,23 | 1.8208 | 0.1904 ns |
| | | ZT 16 | 1,23 | 0.1385 | 0.7131 ns | | | CT 16 | 1,23 | 0.037 | 0.8491 ns |
| | | ZT 17 | 1,23 | 0.1599 | 0.6930 ns | | | ***CT 17*** | 1,23 | 7.9341 | 0.0098 ** |
| | | ZT 18 | 1,23 | 1.1594 | 0.2928 ns | | | CT 18 | 1,23 | 1.7095 | 0.2040 ns |
| | | ZT 19 | 1,23 | 0.3922 | 0.5373 ns | | | CT 19 | 1,23 | 0.5454 | 0.4677 ns |
| | | ZT 20 | 1,23 | 0.3095 | 0.5834 ns | | | CT 20 | 1,23 | 2.8038 | 0.1076 ns |
| | | ZT 21 | 1,23 | 0.3806 | 0.5434 ns | | | CT 21 | 1,23 | 0.4471 | 0.5104 ns |
| | | ZT 22 | 1,23 | 1.562 | 0.2239 ns | | | CT 22 | 1,23 | 0.777 | 0.3872 ns |
| | | ZT 23 | 1,23 | 2.5531 | 0.1237 ns | | | ***CT 23*** | 1,23 | 6.314 | 0.0194 * |
| | LD2 | ***ZT 0*** | 1,23 | 6.4331 | 0.0184 * | | DD2 | ***CT 0*** | 1,23 | 36.5261 | < 0.00001 *** |
| | | ***ZT 1*** | 1,23 | 12.6033 | 0.0017 ** | | | ***CT 1*** | 1,23 | 22.2991 | 0.0001 *** |
| | | ZT 2 | 1,23 | 3.2578 | 0.0842 ns | | | CT 2 | 1,23 | 1.3733 | 0.2532 ns |
| | | ZT 3 | 1,23 | 0.0166 | 0.8986 ns | | | CT 3 | 1,23 | 0.1712 | 0.6829 ns |
| | | ZT 4 | 1,23 | 1.6022 | 0.2183 ns | | | CT 4 | 1,23 | 1.0201 | 0.3230 ns |
| | | ZT 5 | 1,23 | 3.1256 | 0.0903 ns | | | CT 5 | 1,23 | 0.1348 | 0.7169 ns |
| | | ZT 6 | 1,23 | 1.2785 | 0.2698 ns | | | CT 6 | 1,23 | 0.5343 | 0.4722 ns |
| | | ZT 7 | 1,23 | 2.6241 | 0.1189 ns | | | CT 7 | 1,23 | 0.04 | 0.8432 ns |
| | | ZT 8 | 1,23 | 0.3938 | 0.5365 ns | | | CT 8 | 1,23 | 1.8318 | 0.1891 ns |
| | | ZT 9 | 1,23 | 0.4032 | 0.5317 ns | | | ***CT 9*** | 1,23 | 4.5717 | 0.0434 * |
| | | ZT 10 | 1,23 | 0.4659 | 0.5017 ns | | | ***CT 10*** | 1,23 | 25.5313 | < 0.00001 *** |
| | | ***ZT 11*** | 1,23 | 5.34 | 0.0302 * | | | ***CT 11*** | 1,23 | 21.9484 | 0.0001 *** |
| | | ***ZT 12*** | 1,23 | 41.6463 | < 0.00001 *** | | | ***CT 12*** | 1,23 | 39.7052 | < 0.00001 *** |
| | | ***ZT 13*** | 1,23 | 5.2637 | 0.0312 * | | | ***CT 13*** | 1,23 | 35.2004 | < 0.00001 *** |
| | | ZT 14 | 1,23 | 0.7312 | 0.4013 ns | | | ***CT 14*** | 1,23 | 34.26 | < 0.00001 *** |
| | | ***ZT 15*** | 1,23 | 4.3865 | 0.0474 * | | | ***CT 15*** | 1,23 | 8.5386 | 0.0077 ** |
| | | ZT 16 | 1,23 | 0.0022 | 0.9633 ns | | | CT 16 | 1,23 | 0.0088 | 0.9262 ns |
| | | ZT 17 | 1,23 | 2.6558 | 0.1168 ns | | | CT 17 | 1,23 | 1.3539 | 0.2565 ns |
| | | ZT 18 | 1,23 | 1.3678 | 0.2542 ns | | | ***CT 18*** | 1,23 | 4.3313 | 0.0487 * |
| | | ZT 19 | 1,23 | 0.1839 | 0.6720 ns | | | CT 19 | 1,23 | 0.1543 | 0.6981 ns |
| | | ZT 20 | 1,23 | 2.6697 | 0.1159 ns | | | CT 20 | 1,23 | 0.0001 | 0.9914 ns |
| | | ZT 21 | 1,23 | 0.6347 | 0.4338 ns | | | ***CT 21*** | 1,23 | 4.7098 | 0.0406 * |
| | | ZT 22 | 1,23 | 0.891 | 0.3550 ns | | | ***CT 22*** | 1,23 | 12.1055 | 0.0020 ** |
| | | ***ZT 23*** | 1,23 | 23.5396 | 0.0001 *** | | | ***CT 23*** | 1,23 | 28.95 | < 0.00001 *** |
| | LD3 | ***ZT 0*** | 1,23 | 74.9206 | < 0.00001 *** | | DD3 | ***CT 0*** | 1,23 | 56.5965 | < 0.00001 *** |
| | | ZT 1 | 1,23 | 0.3162 | 0.5793 ns | | | ***CT 1*** | 1,23 | 10.2741 | 0.0039 ** |
| | | ZT 2 | 1,23 | 0.3542 | 0.5575 ns | | | CT 2 | 1,23 | 2.3387 | 0.1398 ns |
| | | ZT 3 | 1,23 | 0.4022 | 0.5322 ns | | | CT 3 | 1,23 | 0.0146 | 0.9050 ns |
| | | ZT 4 | 1,23 | 0.6655 | 0.4230 ns | | | CT 4 | 1,23 | 0.3703 | 0.5488 ns |
| | | ZT 5 | 1,23 | 1.2433 | 0.2763 ns | | | CT 5 | 1,23 | 0.2603 | 0.6148 ns |
| | | ZT 6 | 1,23 | 0.832 | 0.3712 ns | | | CT 6 | 1,23 | 0.0035 | 0.9535 ns |
| | | ZT 7 | 1,23 | 0.1265 | 0.7253 ns | | | CT 7 | 1,23 | 3.0746 | 0.0928 ns |
| | | ZT 8 | 1,23 | 2.0496 | 0.1657 ns | | | ***CT 8*** | 1,23 | 8.3143 | 0.0084 ** |
| | | ZT 9 | 1,23 | 1.748 | 0.1991 ns | | | ***CT 9*** | 1,23 | 20.4617 | 0.0002 *** |
| | | ZT 10 | 1,23 | 3.7926 | 0.0638 ns | | | ***CT 10*** | 1,23 | 12.4519 | 0.0018 ** |
| | | ***ZT 11*** | 1,23 | 10.9634 | 0.0030 ** | | | ***CT 11*** | 1,23 | 11.6887 | 0.0023 ** |
| | | ***ZT 12*** | 1,23 | 17.411 | 0.0004 *** | | | ***CT 12*** | 1,23 | 32.1227 | < 0.00001 *** |
| | | ***ZT 13*** | 1,23 | 5.0828 | 0.0340 * | | | ***CT 13*** | 1,23 | 99.6479 | < 0.00001 *** |
| | | ZT 14 | 1,23 | 2.4819 | 0.1288 ns | | | ***CT 14*** | 1,23 | 27.134 | < 0.00001 *** |
| | | ZT 15 | 1,23 | 0.169 | 0.6848 ns | | | CT 15 | 1,23 | 1.9002 | 0.1813 ns |
| | | ZT 16 | 1,23 | 0.7347 | 0.4002 ns | | | CT 16 | 1,23 | 0.2811 | 0.6010 ns |
| | | ZT 17 | 1,23 | 0.2798 | 0.6019 ns | | | CT 17 | 1,23 | 0.8634 | 0.3624 ns |
| | | ***ZT 18*** | 1,23 | 4.3015 | 0.0495 * | | | CT 18 | 1,23 | 1.5524 | 0.2253 ns |
| | | ZT 19 | 1,23 | 2.0099 | 0.1697 ns | | | CT 19 | 1,23 | 0.0506 | 0.8241 ns |
| | | ZT 20 | 1,23 | 1.5945 | 0.2193 ns | | | CT 20 | 1,23 | 0.1444 | 0.7074 ns |
| | | ***ZT 21*** | 1,23 | 6.9371 | 0.0148 * | | | CT 21 | 1,23 | 0.4954 | 0.4886 ns |
| | | ***ZT 22*** | 1,23 | 16.5428 | 0.0005 *** | | | ***CT 22*** | 1,23 | 10.3402 | 0.0038 ** |
| | | ***ZT 23*** | 1,23 | 19.3617 | 0.0002 *** | | | ***CT 23*** | 1,23 | 5.7768 | 0.0247 * |

A two-way repeated ANOVA was performed followed by simle main effect test (Table 1). Multiple comparisons were used for average of total sleep at each time through LD and DD condition respectly and were performed to determine when were significantly different. *p < .005, **p < 0.01, ***p < 0.001
