# Supplementary material for: A phosphorylation-deficient mutant of Sik3, a homolog of Sleepy, alters circadian sleep regulation by PDF neurons in Drosophila
Source: Front Neurosci. 2023 Aug 17;17:1181555. doi: 10.3389/fnins.2023.1181555 (PMC10469759; doi:10.3389/fnins.2023.1181555)
Supplement: Supplementary file 2 [file Data_Sheet_2.PDF]

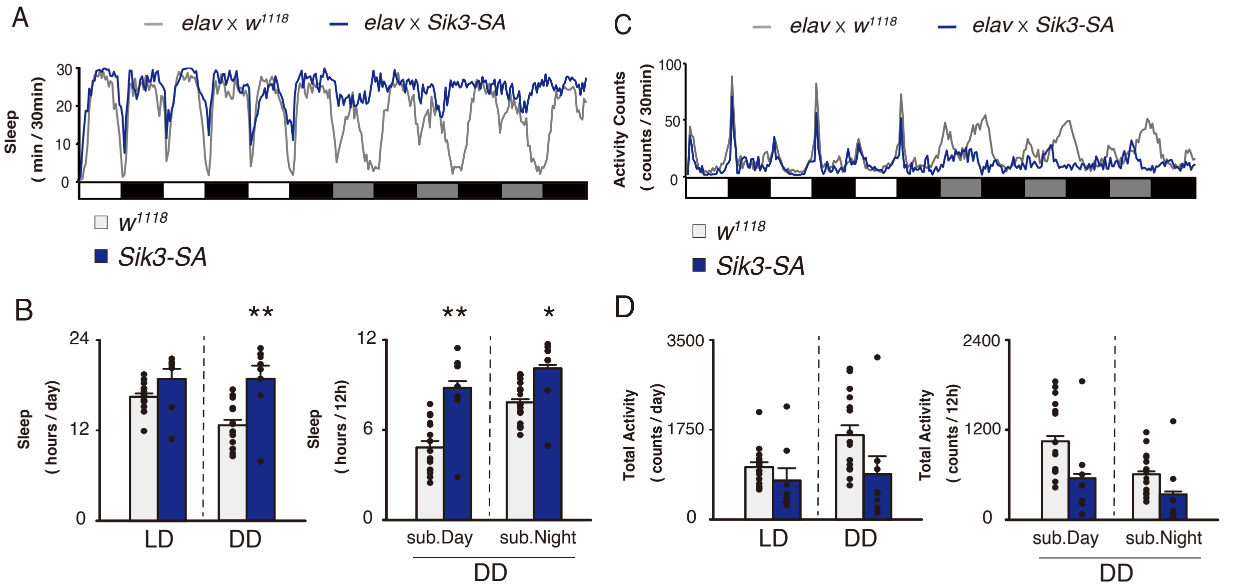


**Kobayashi et al., Figure S1**

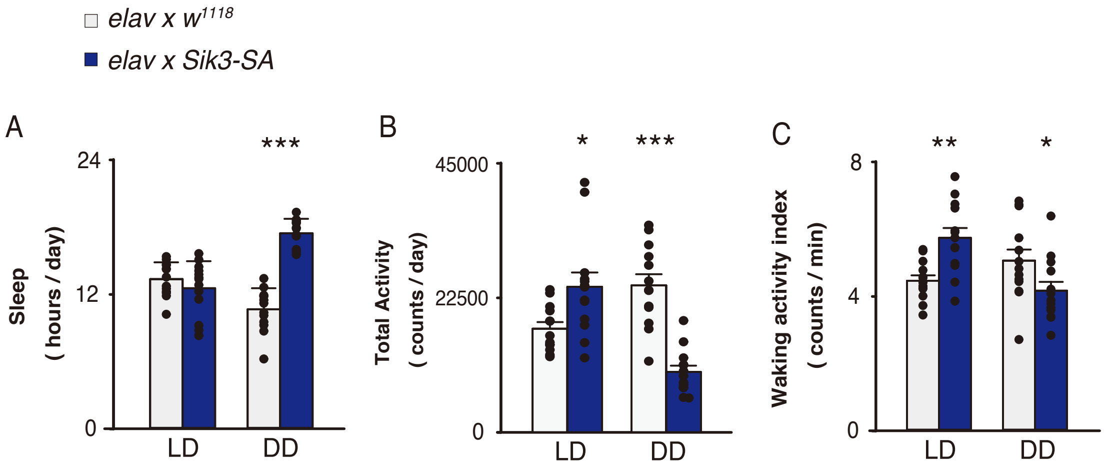


***Kobayashi et al., Figure S2***

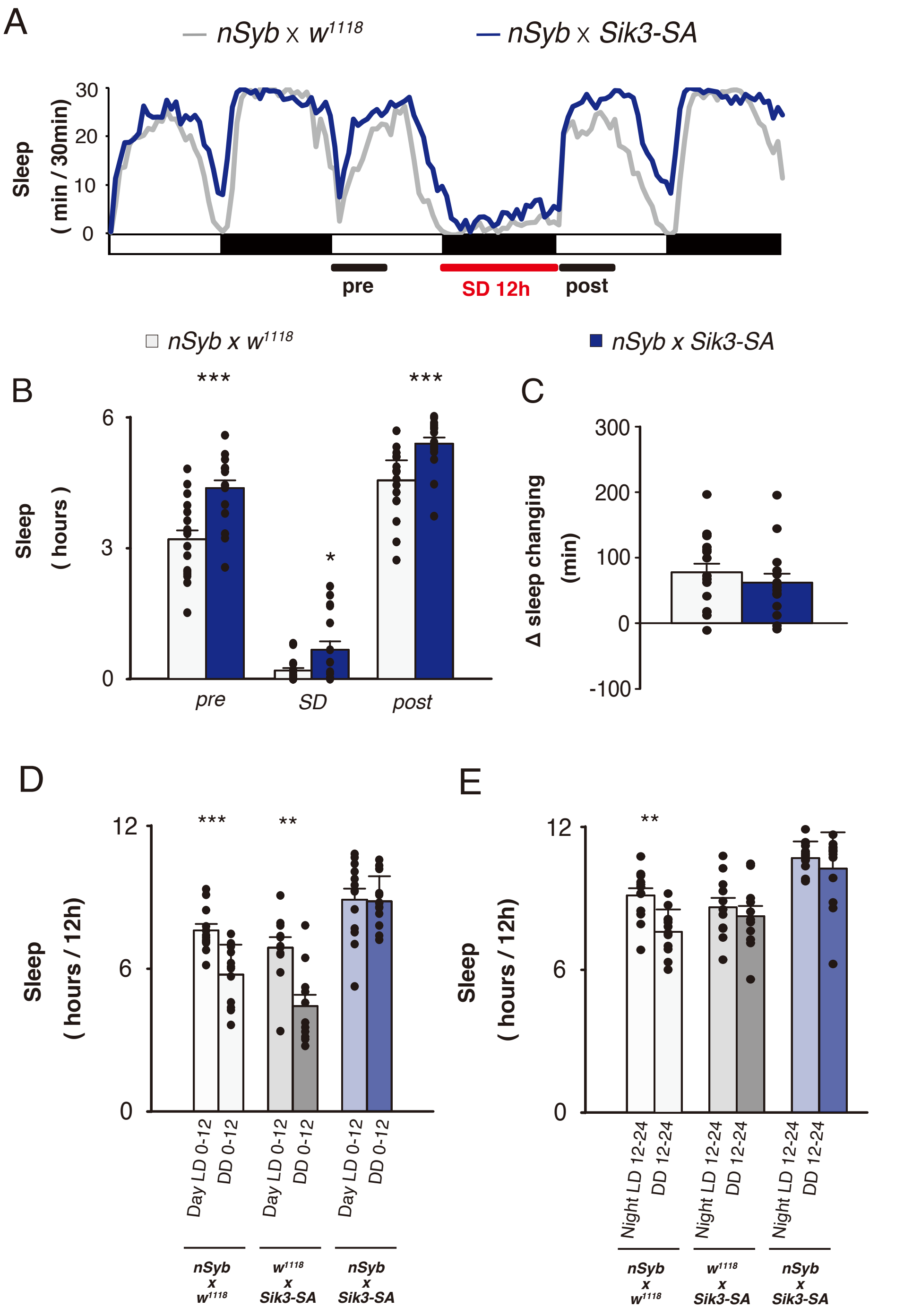


**Kobayashi et al., Figure S3**

A *nSyb x w*[1118]

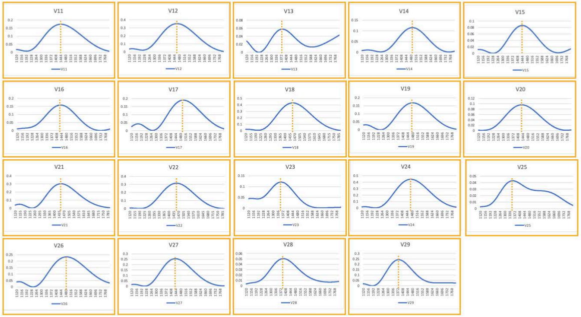


B *w*[1118] *x Sik3-SA*

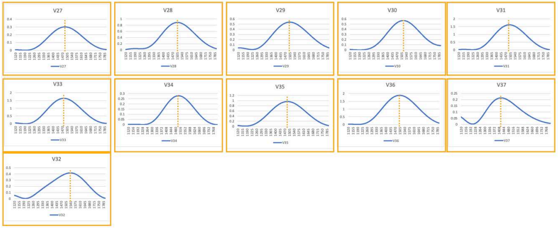


C *nSyb x Sik3-SA*

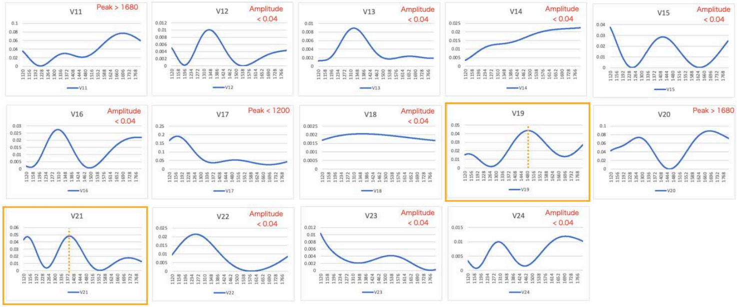


**Kobayashi et al., Figure S4**

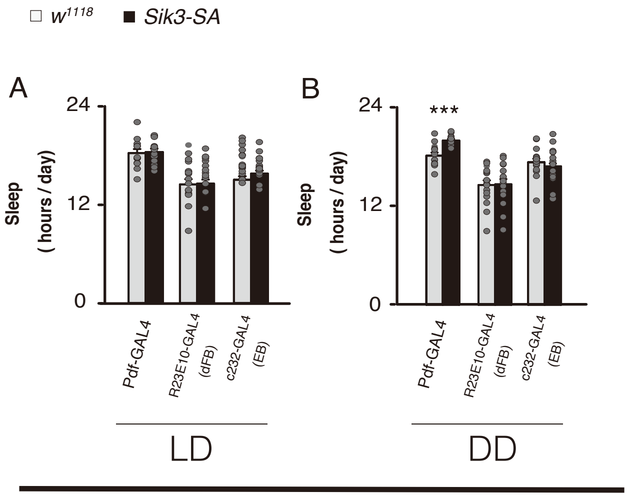


# *Kobayashi et al., Figure S5*

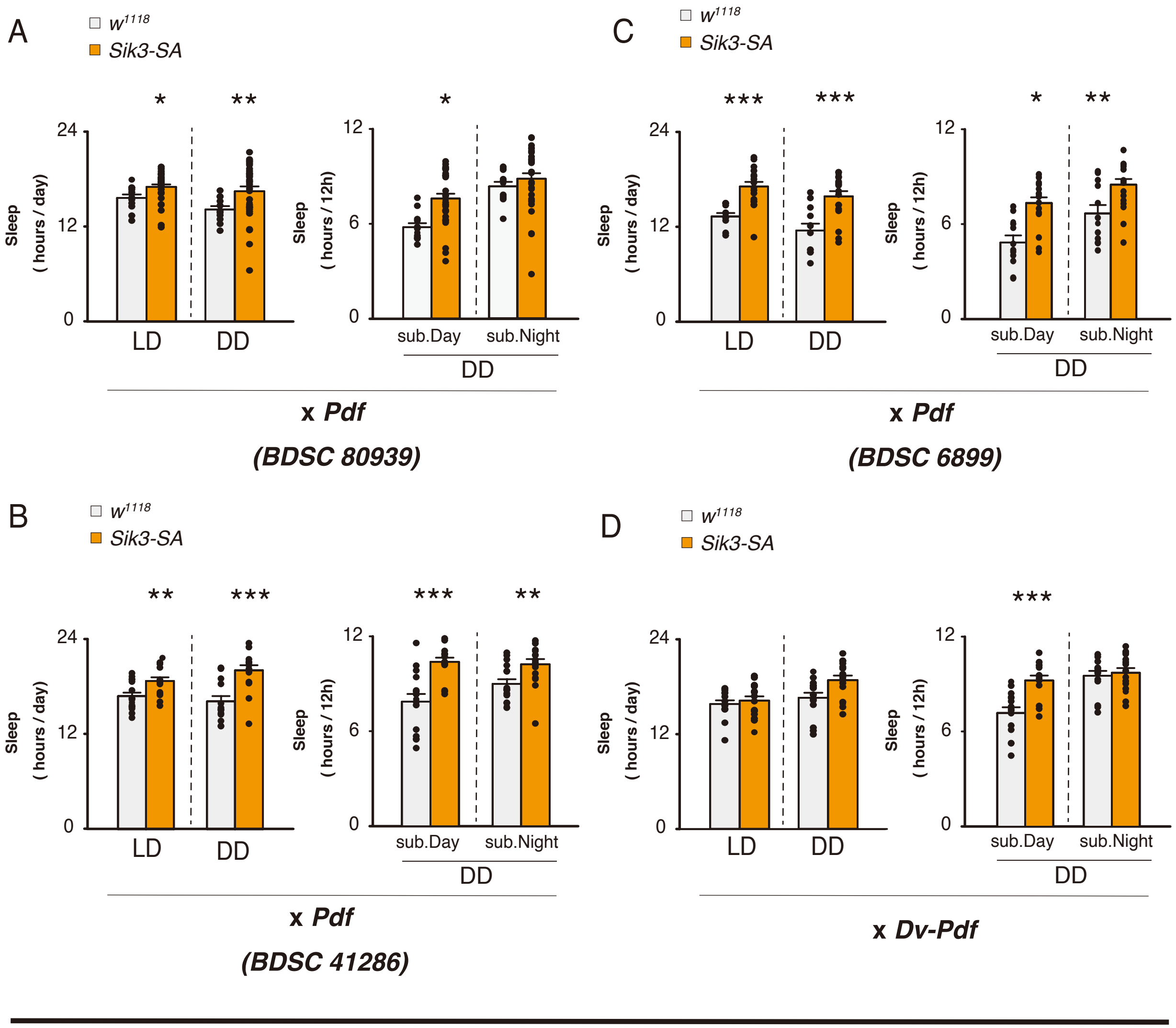


**Kobayashi et al., Figure S6**

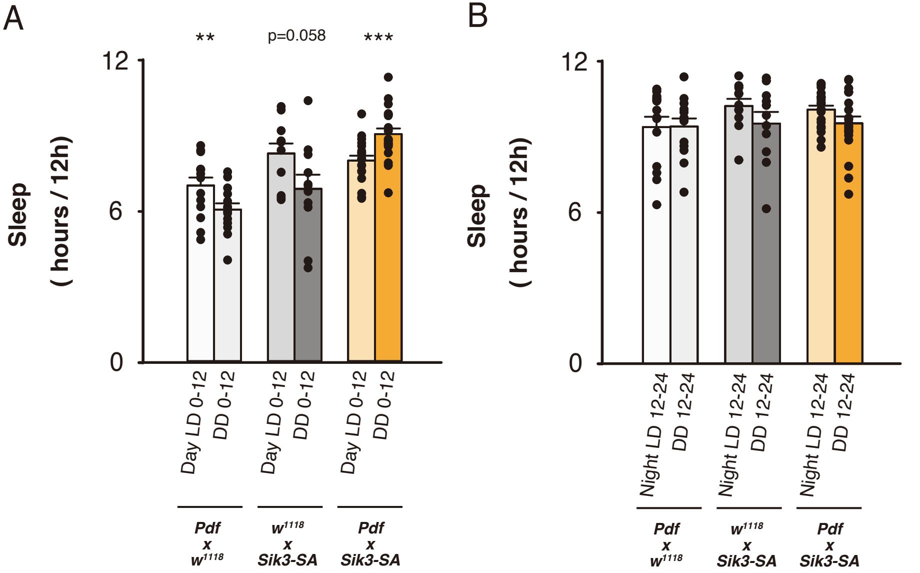


***Kobayashi et al., Figure S7***

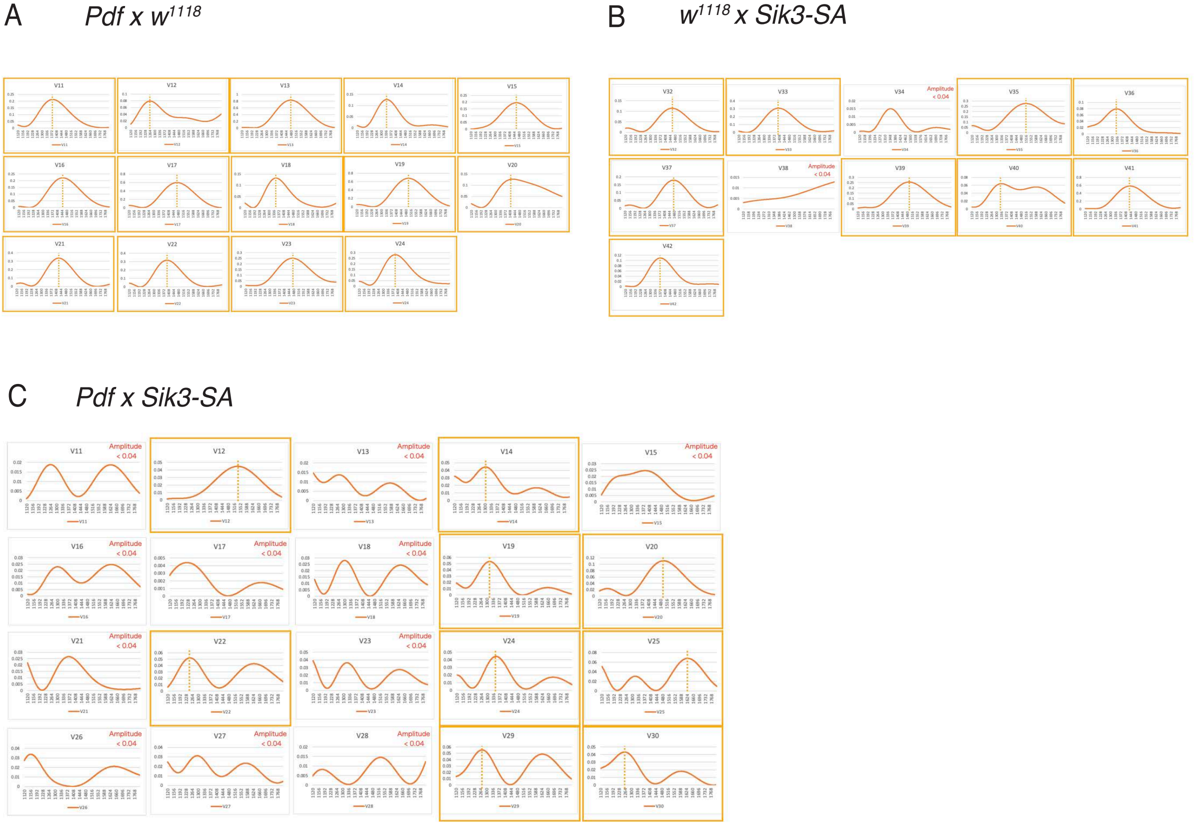


**Kobayashi et al., Figure S8**

A *nSyb x w[1118]*

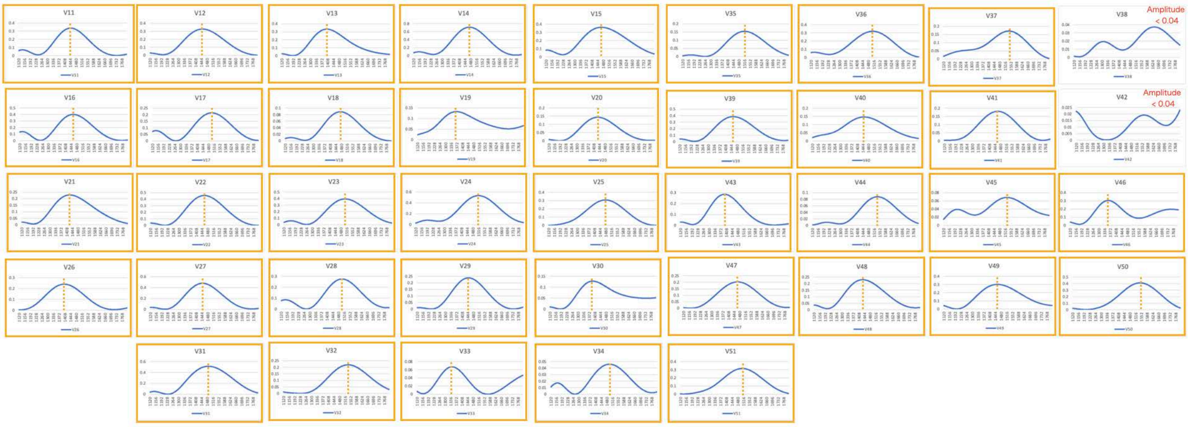


B *nSyb x Pdf-GAL80*

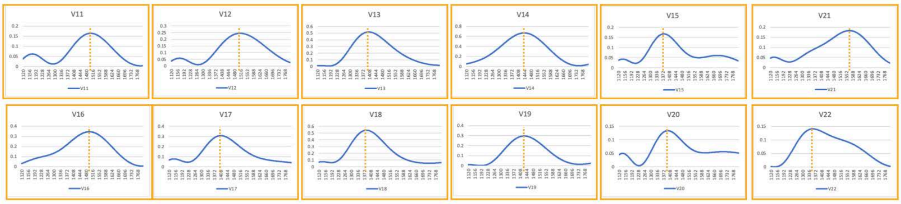

C *nSyb x Sik3-SA*

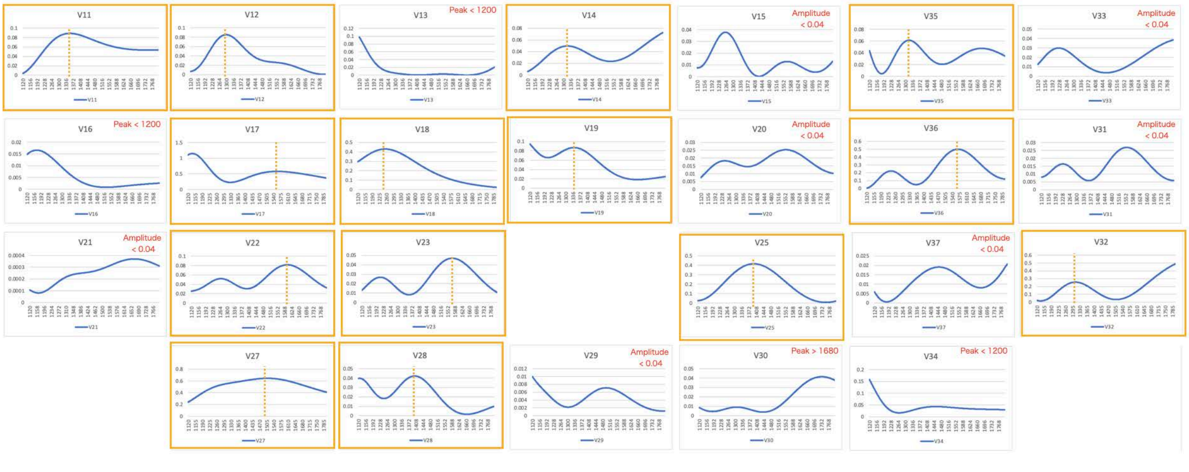


D *nSyb x Pdf-GAL80, Sik3-SA*

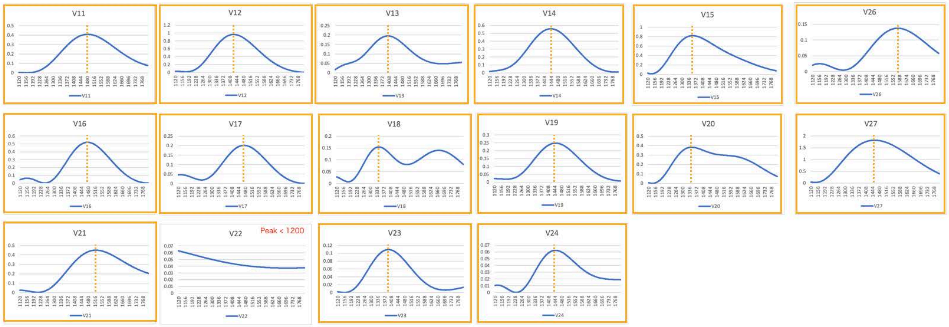


***Kobayashi et al., Figure S9***

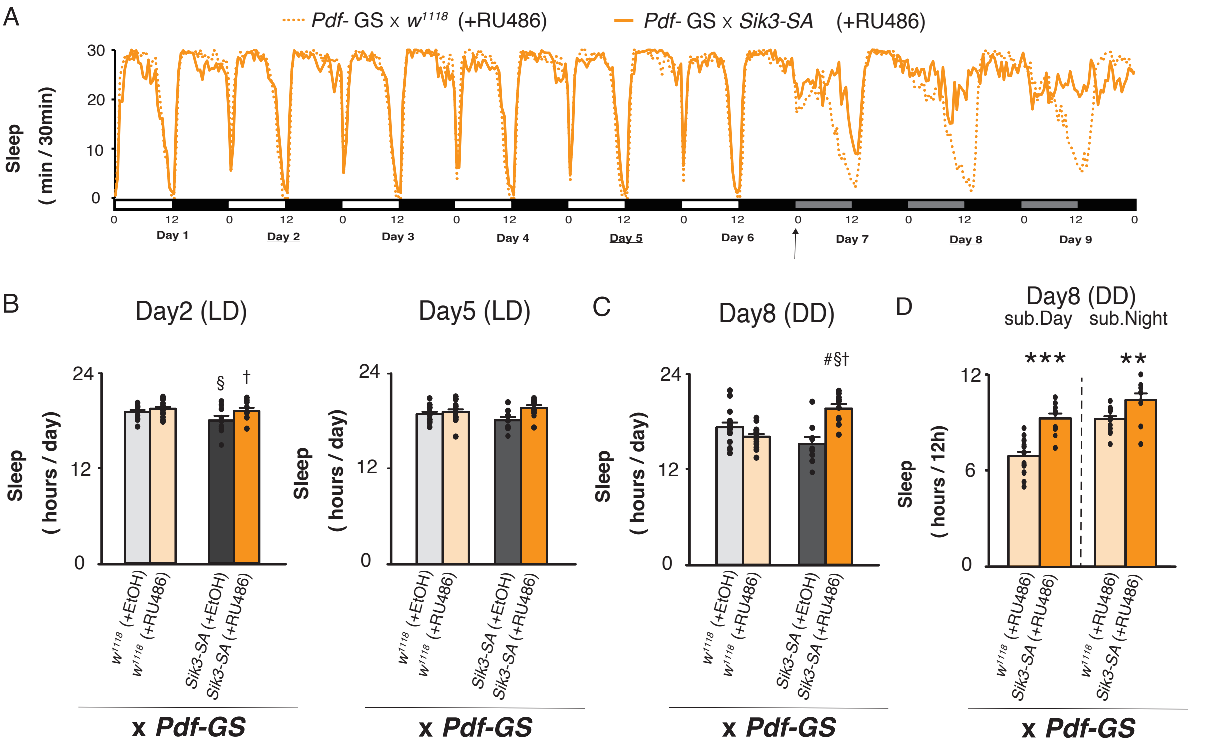


Kobayashi et al., Figure S10

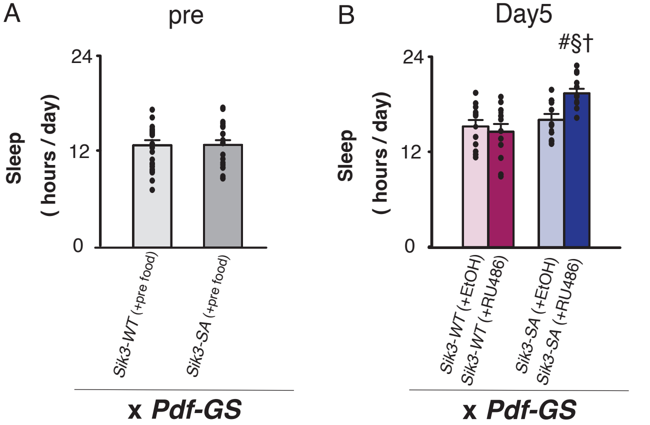


# *Kobayashi et al., Figure S11*
