# Supplementary material for: A phosphorylation-deficient mutant of Sik3, a homolog of Sleepy, alters circadian sleep regulation by PDF neurons in Drosophila
Source: Front Neurosci. 2023 Aug 17;17:1181555. doi: 10.3389/fnins.2023.1181555 (PMC10469759; doi:10.3389/fnins.2023.1181555)
Supplement: Supplementary file 3 [file Data_Sheet_3.pdf]

Supplementary Figure Legends

Figure S1. Pan-neuronal *Sik3-SA* overexpression by *elav-GAL4*

(A) Average sleep profiles for 30-min intervals of control flies (*elav-GAL4* × w^{1118}, gray line, n = 16) and flies with the pan-neuronal overexpression of *Sik3-SA* (*elav-GAL4* × *UAS-Sik3-SA*, blue line, n = 8). Each fly was recorded for 3 days under LD followed by 3 days under constant dark (DD) conditions. Day and night under LD and subjective daytime (DD 0-12) and nighttime (DD 12-24) under DD condition are depicted by white, gray and black bars, respectively. (B) Total daily sleep of *elav-GAL4* × w^{1118} (gray bar) and *elav-GAL4* × *UAS-Sik3-SA* (blue bar) flies under LD and DD (left) and during subjective daytime and nighttime under DD condition (right). (C) Average active counts profiles for 30-min intervals of control flies (*elav-GAL4* × w^{1118}, gray line, n =16) and flies with the pan-neuronal expression of *Sik3-SA* (*elav-GAL4* × *UAS-Sik3-SA*, blue line, n = 8). (D) Total activity counts of *elav*-GAL4 x w^{1118} (gray bar) and *elav*-GAL4 × *UAS-Sik3-SA* (blue bar) flies under LD and DD condition (left) and during subjective daytime and nighttime under DD condition (right). Data are presented as mean ± SEM. ∗∗ $p < 0.01$, ∗ $p < 0.05$; Welch's t-test.

Figure S2. Multibeam monitor analysis of flies with pan-neuronal *Sik3-SA* overexpression.

(A) Total daily sleep of *elav-GAL4* × w^{1118} (gray bar, n=13) and *elav-GAL4* × *UAS-Sik3-SA* (blue bar, n=13) flies under LD and DD with MB5, multibeam activity monitor. (B) Total activity counts of *elav-GAL4* × w^{1118} (gray bar) and *elav-GAL4* × *UAS-Sik3-SA* (blue bar) flies under LD and DD with MB5. (C) Waking activity index of *elav-GAL4* × w^{1118} (gray bar) and *elav-GAL4* × *UAS-Sik3-SA* (blue bar) flies under LD and DD with MB5. Data are presented as mean ± SEM. ∗∗∗ $p < 0.001$, ∗∗ $p < 0.01$, ∗ $p < 0.05$; Welch's t-test.

Figure S3. Sleep deprivation and comparison of sleep between day and night in flies with pan-neuronal *Sik3-SA* overexpression.

(A) Average sleep profiles for 30-min intervals of control flies (*nSyb-GAL4* × w^{1118}, gray line, n = 17) and flies with the pan-neuronal overexpression of *Sik3-SA* (*nSyb-GAL4* × *UAS-Sik3-SA*, blue line, n = 15). Each fly was recorded for 3 days under LD and deprived sleep by mechanical stimulation during 12 hours on the night of the 2nd day. Bars under the graph depicted the periods of sleep measurements and sleep deprivation (red). (B) Sleep amount of *nSyb-GAL4* × w^{1118} (gray bar) and *nSyb-GAL4* × *UAS-Sik3-SA* (blue bar) flies during pre (6 hours), SD (12 hours) and post (6 hours). (C) Sleep duration comparing between pre and post period of (B). (D) Total daily sleep of *nSyb-GAL4* ×

w^{1118} (light gray bar, n=19), w^{1118} × *UAS-Sik3-SA* (dark gray bar, n=11) and *nSyb-GAL4* × *UAS-Sik3-SA* (blue bar, n=13). Each group was compared between day time on and DD 0-12. (E) Total daily sleep of *nSyb-GAL4* × w^{1118} (light gray bar, n=19), w^{1118} × *UAS-Sik3-SA* (dark gray bar, n=11) and *nSyb-GAL4* × *UAS-Sik3-SA* (blue bar, n=14). Each group was compared between night time and DD 12-24. Data are presented as mean ± SEM. ∗∗∗ $p < 0.001$, ∗∗ $p < 0.01$; Welch's t-test.

Figure S4. Power spectrum data obtained through FFT analysis in flies with pan-neuronal overexpression of *Sik3-SA*.

5 days behavioral results under DD condition (the same results as Figure 1) were applied to FFT power analysis and each graph showed flies' spectrum pattern. Control groups (A; *nSyb-GAL4* × w^{1118}, n = 19 and B; w^{1118} × *UAS-Sik3-SA,* n=11) showed that only one peak located between 1300-1400 min, on the other hand, pan-neuronal overexpression of *Sik3-SA* (C; *nSyb-GAL4* × *UAS-Sik3-SA*, n=14) showed low amplitude peak or doubled peaks. Flies squared by yellow line were calculated their circadian period length because their amplitude were over 0.04 and their peaks were located between 1200-1580 mins.

Figure S5. The effects of *Sik3-SA* overexpression using region-specific *GAL4* drivers.

Total sleep amount of control flies (white bar) and flies with overexpression of *Sik3-SA* by the following *GAL4* drivers (black bar) under LD (A) and DD (B); *Pdf-GAL4* (n= 13 and 13), *R23E10-GAL4* (n= 16 and 16), *c232-GAL4* and (n= 16 and 16). The experimental conditions were same as in Figure S1. Data are presented as mean ± SEM. ∗∗∗ $p < 0.001$; Welch's t-test.

Figure S6. The effects of *Sik3-SA* overexpression using different *GAL4* drivers specific to PDF neurons.

(A-D) Total daily sleep of control *Pdf-GAL4* drivers × w^{1118} flies (gray bar) and flies overexpressing *Sik3-SA* by *Pdf-GAL4* drivers; (A) BDSC 80939 (n=12, 32), (B) BDSC 41286 (n=16, 16), (C) BDSC 6899 (n=12, 18) and (D) *DvPdf-GAL4* (n=15, 16) (orange bar) under LD and DD condition (left) and during subjective day and nighttime under DD condition (right). The experimental conditions were the same as in Figure S1. Data are presented as mean ± SEM. ∗∗∗ $p < 0.001$, ∗∗ $p < 0.01$, ∗ $p < 0.05$; Welch's t-test.

Figure S7. The effect of light condition change on sleep increase induced by PDF specific *Sik3-SA*

overexpression.

(A) Total daily sleep of *Pdf-GAL4* × w^{1118} (gray bar, n=14), w^{1118} × *Sik3-SA* (dark gray bar, n=10), and *Pdf-GAL4* × *UAS-Sik3-SA* (orange bar, n=20). Each group was compared between day time on and DD 0-12. (B) Total daily sleep of *Pdf-GAL4* × w^{1118} (gray bar, n=14), w^{1118} × *Sik3-SA* (dark gray bar, n=10), and *Pdf-GAL4* × *UAS-Sik3-SA* (orange bar, n=20). Each group was compared between night time and DD 12-24. Data are presented as mean ± SEM. ∗∗∗ $p < 0.001$, ∗∗ $p < 0.01$; Welch's t-test.

Figure S8. Power spectrum data obtained through FFT analysis in flies overexpressing *Sik3-SA* specifically in PDF neurons.

5 days behavioral results under DD condition (the same results as Figure 2) were applied to FFT power analysis and each graph showed flies' spectrum pattern. Almost all control groups (A; *Pdf-GAL4* × w^{1118}, n = 14 and B; w^{1118} × *UAS-Sik3-SA,* n=11) showed that only one peak located between 1300-1400 min, on the other hand overexpressed *Sik3-SA* in PDF neurons (C; *Pdf-GAL4* × *UAS-Sik3-SA*, n=20) showed low amplitude peak or doubled peaks. Flies squared by yellow line were calculated their circadian period length because their amplitude were over 0.04 and their peaks were located between 1200-1580 mins.

Figure S9. Power spectrum data obtained through FFT analysis in flies overexpressing *Sik3-SA* in all neurons excluding PDF neurons.

5 days behavioral results under DD condition (the same results as Figure 3) were applied to FFT power analysis and each graph showed flies' spectrum pattern. Control groups (A; *nSyb-GAL4* × w^{1118}, n = 41 and B; *nSyb-GAL4* × *Pdf-GAL80,* n=12) showed that only one peak located between 1300-1400 min and as shown in Figure S4, pan-neuronal overexpression of *Sik3-SA* (C; *nSyb-GAL4* × *UAS-Sik3-SA*, n=25) showed low amplitude peak or doubled peaks. Inhibition of overexpression of *Sik3-SA* in PDF neurons by *Pdf-GAL80* (D; *nSyb-GAL4* × *Pdf-GAL80*; *UAS-Sik3-SA*, n=14) was normalized their FFT power spectrum patterns. Flies squared by yellow line were calculated their circadian period length because their amplitude were over 0.04 and their peaks were located between 1200-1580 mins.

Figure S10. The increase in sleep observed with adult-specific overexpression of *Sik3-SA* in PDF neurons.

(A) Average sleep profiles for 30-min intervals of control flies (*Pdf*-GS × w^{1118} (+RU486), orange dotted lines, n = 15) and flies with the conditional overexpression of *Sik3-SA* in PDF neurons (*Pdf*-GS × UAS-*Sik3-SA* (+RU486),

orange lines, n = 9) for 6 days under LD conditions followed 3 days under DD conditions. (B) Total daily sleep of control flies (*Pdf*-GS × w^{1118} (+EtOH), pale gray bar, n = 15; *Pdf*-GS × w^{1118} (+RU486), pale orange bar, n = 16; and *Pdf*-GS × UAS-*Sik3-SA* (+EtOH), dark gray bar, n = 9)) and flies with the conditional overexpression of *Sik3-SA* in PDF neurons *(Pdf*-GS × UAS-*Sik3-SA* (+RU486), dark orange bar, n = 9) on Day 2 and 5 under LD condition. (C) Total daily sleep of control flies (*Pdf*-GS × w^{1118} (+EtOH), pale gray bar, n = 15; *Pdf*-GS × w^{1118} (+RU486), pale orange bar, n = 16; and *Pdf*-GS × UAS-*Sik3-SA* (+EtOH), dark gray bar, n = 9)) and flies with the conditional overexpression of *Sik3-SA* in PDF neurons *(Pdf*-GS × UAS-*Sik3-SA* (+RU486), dark orange bar, n = 9) on Day 8 under DD conditions. (D) Total daily sleep of control flies (*Pdf*-GS × w^{1118} (+RU486), pale orange bar, n = 16) and flies with the conditional overexpression of *Sik3-SA* in PDF neurons *(Pdf*-GS × UAS-*Sik3-SA* (+RU486), dark orange bar, n = 9) under subjective day and nighttime on Day 8 under DD conditions. Data are presented as mean ± SEM. # $p < 0.05$ vs. *Pdf*-GS × w^{1118} (+EtOH), § $p < 0.05$ vs. *Pdf*-GS × w^{1118} (+RU486), † $p < 0.05$ vs. *Pdf*-GS × *UAS-Sik3-SA* (+EtOH); Tukey-Kramer method. *** $p < 0.001$, ** $p < 0.01$; Welch's t-test.

Figure S11. Adult-specific overexpression of *Sik3-WT* and *Sik3-SA* in all neurons and PDF neurons.

(A) Total daily sleep of flies with conditional overexpression of *Sik3* (*Pdf*-GS × w^{1118} (pre-food (5% sucrose and 1% agar)), pale gray bar, n = 24) and *Sik3-SA* in all neurons (*Pdf*-GS × *UAS-Sik3-SA* (pre-food), dark gray bar, n = 22) in pre-period. (B) Total daily sleep of control flies (*Elav*-GS× *Sik3-WT* (+EtOH), pale purple bar, n = 12 and *Elav*-GS × UAS-*Sik3-SA* (+EtOH), pale blue bar, n = 11) and flies overexpression of *Sik3* (*Elav*-GS × UAS-*Sik3-WT* (+RU486), dark purple bar, n = 12) and of *Sik3-SA* in all neurons (*Elav*-GS × UAS-*Sik3-SA* (+RU486), dark blue bar, n = 11) on Day 5 under DD condition. Data are presented as mean ± SEM. # $p < 0.05$ vs. *Elav*-GS × *Sik3-WT* (+EtOH), § $p < 0.05$ vs. *Elav*-GS × *UAS-Sik3-WT* (+RU486), † $p < 0.05$ vs. *Pdf*-GS × *UAS-Sik3-SA* (+EtOH); Tukey-Kramer method.

Figure S12. Daily expression pattern of clock genes.

(A, B) Relative expression patterns of circadian clock genes (*per, tim, Clk*) in the heads of *nSyb-GAL4* x w^{1118} and *nSyb-GAL4* x *UAS-Sik3-SA* flies denoted at the bottom of each panel under LD (A) and DD (B) conditions. Error bars indicate SEM.
